# Knockout of Low‐Density Lipoprotein Receptor‐Related Protein 1 From Astrocytes in Adult Mice Accelerates Long‐Term Functional Recovery After Ischemic Stroke

**DOI:** 10.1002/brb3.71300

**Published:** 2026-03-31

**Authors:** Meng Wang, Sadiya N. Ahmad, Pamela Reed, Shane Sprague, Naomi L. Sayre

**Affiliations:** ^1^ Department of Neurosurgery University of Texas Health Science Center San Antonio Texas USA; ^2^ South Texas Medical Scientist Training Program University of Texas Health Science Center San Antonio Texas USA; ^3^ South Texas Veteran's Health Care System San Antonio Texas USA

**Keywords:** astrocyte, gliosis, LRP1, middle cerebral artery occlusion, recovery, stroke

## Abstract

**Purpose:**

Ischemic stroke is a primary cause of death and disability worldwide; however, therapeutic opportunities are limited. Astrocytes, a major class of caretaker glia in the brain, can significantly alter outcomes after stroke through multiple pathways. Low‐density lipoprotein receptor‐related protein 1 (LRP1) is a multifunctional receptor that can modulate cellular signaling through its interaction with a diverse array of signaling mediators; however, its role in astrocyte function is not well elucidated. We tested whether LRP1 in astrocytes could alter outcomes in both the acute phase (24 h) and chronic phase (3–9 months) after middle cerebral artery occlusion in mice.

**Methods:**

Astrocyte‐specific *Lrp1* knockout mice were generated by crossing *Cx30‐CreER^T2^* mice with *Lrp1*‐floxed mice. As controls, mice were compared to *Cx30‐CreER^T2^* mice with wild‐type *Lrp1*. Cre activation was induced by tamoxifen treatment at 2 months of age in all mice. At 3 months of age, male and female mice were subjected to either middle cerebral artery occlusion for 1 h or sham surgery. Mice underwent motor coordination testing, and tissues were harvested at 24 h, 7 days, 3 months, or 9 months post‐surgery for subsequent histological analysis.

**Findings:**

We found that genetic knockout of *Lrp1* in astrocytes worsened motor coordination in mice acutely after middle cerebral artery occlusion, but paradoxically improved long‐term outcomes by 3 months after stroke. Notably, at 3 months post‐stroke, loss of astrocyte LRP1 was associated with improved motor outcomes and reduced gliosis.

**Conclusion:**

Our results suggest that loss of astrocyte LRP1 accelerates recovery after ischemic stroke.

## Graphical abstract

We find that loss of LRP1 in astrocytes improves long‐term outcomes after ischemic stroke and is associated with reduced gliosis. Our findings suggest a role for LRP1 in astrocyte reactivity and response to stroke.

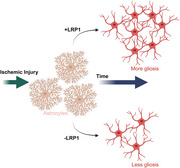

## 1 Introduction

Stroke, a leading cause of disability and death worldwide, presents a significant challenge in medical care due to its complex pathophysiology and recovery mechanisms. Ischemic stroke, which accounts for approximately 87% of all stroke cases, is caused by a blockage in a blood vessel supplying the brain (Tsao et al. 2023). This blockage results in a lack of blood flow, leading to neuronal death and severe functional impairments. In the acute phase, stroke can cause paralysis, speech difficulties, and cognitive decline. Long‐term effects often include motor dysfunction, memory problems, and emotional disturbances, which can persist long after the initial blockage has resolved. These lasting impacts of ischemic stroke significantly affect the quality of life of patients and their families. Therefore, identifying biological targets that influence long‐term recovery after ischemic stroke is crucial for developing effective therapies.

Emerging evidence suggests that glial cells play a significant role in both the acute injury phase and long‐term recovery processes. Astrocytes, the most abundant glial cells in the brain, are crucial for supporting neuronal function and maintaining the blood–brain barrier. After brain injury such as ischemic stroke, astrocytes undergo significant changes in morphology and function, a process known as reactive astrogliosis (Liddelow et al. 2017). In recent years, greater appreciation has been given to divergent gene expression, functionality, and morphology among individual astrocytes (Matias et al. 2019), and similarly, astrocyte reactivity is also heterogeneous (Escartin et al. 2021), affecting injury outcomes in complex ways, which can be both beneficial and harmful (Li et al. 2022). Thus, understanding the factors that influence astrocyte reactivity and heterogeneity will enable more nuanced targeting and enhanced therapeutics that stimulate the beneficial effects of astrocyte function while limiting harmful effects.

Reactive astrogliosis is modulated by various molecular pathways, including inflammatory stimulation (Balasingam et al. 1994; Sriram et al. 2004), calcium signaling (Kanemaru et al. 2013), histone modulation (Clayton et al. 2024), and others (Pekny and Pekna 2016); however, the understanding of factors that influence astrocyte reactivity, especially after ischemia, is still emerging. One factor which could be involved in astrocyte reactivity is low‐density lipoprotein receptor‐related protein 1 (LRP1), a transmembrane receptor involved in endocytosis and signal transduction (Mao et al. 2017) and has been implicated in the modulation of pro‐inflammatory pathways (Gaultier et al. 2008; Kawamura et al. 2007; Moynagh 2005). Our current understanding of LRP1 on astrocyte functionality and reactivity is limited, particularly regarding outcomes after ischemic stroke. LRP1 expression is altered in vivo after ischemia (Yamada et al. 2019), and at least one potential risk variant for stroke has been identified (Harriott et al. 2015). We sought to better understand the effect of astrocyte LRP1 on outcomes after ischemic stroke. Adult mice were subjected to inducible deletion of LRP1 in connexin‐30–expressing astrocytes at 2 months of age, and then were given middle cerebral artery occlusion (MCAO) or sham surgery at 3 months. Mice were harvested at 24 h, 7 days, 3 months, or 9 months after surgery to test the effect on acute and long‐term outcomes. Here, we report that while mice lacking astrocyte LRP1 had worsened acute recovery 24 h post‐MCAO, mice had improved behavioral outcomes and reduced gliosis at 3 months post‐MCAO. Our findings suggest that reducing astrocyte LRP1 function could be a chronic therapeutic strategy to mitigate long‐term negative effects due to ischemic stroke.

## 2 Materials and Methods

### 2.1 Animals

All mouse procedures were approved by the University of Texas Health San Antonio Institutional Animal Care and Use Committee (IACUC) in accordance with NIH guidelines. Mice were bred in the vivarium under standard housing conditions with a 12 h light/dark cycle and ad libitum access to food and water*. Connexin 30‐CreER^T2^ (Cx30‐Cre)* mice were a generous gift from Dr. Frank Pfrieger (Slezak et al. 2007) (European Neuroscience Institute, Strasbourg, France) under a material transfer agreement to Dr. James Lechleiter (UT Health San Antonio). Astrocyte‐specific **LRP1KO** mice were generated by crossing: *Cx30‐CreER^T2^* for astrocyte‐specific Cre recombinase expression, and *Lrp1^fl/fl^* mice (B6;129S7‐Lrp1^tm2Her^/J, Jackson stock 012604; Rohlmann et al. 1996). Cousins expressing *Cx30‐CreER^T2^* and wild‐type *Lrp1* (*Lrp1^+/+^*) were used as **Control**. LRP1KO mice were also crossed with *stop‐floxed tdTomato* Ai14 reporter mice (*stop^fl/fl^‐tdTomato*; Jackson stock 007914) to generate **tomato‐LRP1KO** mice. Similarly, cousins expressing *Lrp1^+/+^* and *stop^fl/fl^‐tdTomato* were used as **tomato‐Control**. To minimize differences from genetic drift, KO and Control mice were only one generation removed. KO was induced at 2 months old with tamoxifen IP injection (100 µL of 20 µg/µL dissolved in corn oil) daily for 5 consecutive days. At the experimental endpoint, mice were euthanized with 5% isoflurane/oxygen before intracardic perfusion with ice‐cold PBS followed by 4% paraformaldehyde (PFA)/PBS.

### 2.2 Transient Middle Cerebral Artery Occlusion (MCAO)

MCAO was performed as previously described (Sayre et al. 2017). Briefly, anesthetized mice (1%–3% isoflurane/oxygen) were maintained on a warming pad, and an intraluminal filament was inserted into the left middle cerebral artery via the left carotid artery using aseptic techniques. Blood flow was monitored using a laser Doppler probe (Perimed) affixed to the skull. The filament remained in place for 1 h before removal. Mice were given buprenorphine (0.05 mg/kg of body weight, IP, twice daily) for 48 h after surgery. Using this protocol, we observed that most mice died within the 1st week after MCAO surgery but then survived thereafter until the endpoint. No sham‐injured mice died during the experiment:

**Table:** 

Genotype	Time point	Total MCAO	Total survived	% Survival
Control	24 h	8	6	75%
LRP1KO	24 h	8	6	75%
Control	7 days	9	6	67%
LRP1KO	7 days	6	4	67%
Control	3 months	7	7	100%
LRP1KO	3 months	8	6	75%
Control	9 months	7	5	71%
LRP1KO	9 months	9	8	89%

### 2.3 Behavioral Testing

All behavioral testing was performed by experimenters blinded to genotype and treatment conditions, and results were analyzed together at the experimental endpoint. Prior to experimental testing, mice were allowed to acclimate to the behavioral testing room for at least 30 min. Tests that occurred more than once (rotarod, open field, and NSS) were performed by the same researcher throughout the duration of the study.

#### 2.3.1 Modified Neurological Severity Score (NSS)

Neurological impairment was measured following previously established NSS protocols (Bieber et al. 2019). Mice without deficits score a 0, while those with the most severe deficits score a 14.

#### 2.3.2 Rotarod

Motor coordination was assessed via the rotarod test. One week before surgery, mice were trained to walk on the rotarod (Four Lane Rotarod; Ugo Basile, Italy, #MSW‐007) over 4 consecutive days, gradually increasing the speed each day. Mice were given 3 training sessions daily, with each session spaced at least 30 min apart. By Day 4, mice were expected to remain on the rotarod at 16 RPM for up to 100 s within one session. During testing, mice were placed on the rotarod, which accelerated from 4 RPM to 40 RPM over 300 s. The latency to fall was recorded for 3 testing sessions, with a 30‐min break between each session. The final measurement was the average of the 2 best trials out of 3 for each mouse.

#### 2.3.3 Open Field

Motor activity was measured via the open field assay. Each mouse was placed into the center of an open field (40 × 40 cm) by a blinded male researcher, and exploration was tracked for 300 s using AnyMaze software (Stoelting). Distance traveled, time in center, rotations, and speed were recorded. Mice were then returned to their home cage and the open field arena was thoroughly cleaned with 70% EtOH between each mouse.

### 2.4 2,3,5‐Triphenyltetrazolium Chloride (TTC) Staining

Lesion areas and volume 24 h after MCAO were determined by TTC staining (Bederson et al. 1986). Brains were harvested from euthanized mice and kept in ice‐cold PBS for 5 min. Brains were then sliced into 1 mm coronal sections with a precision brain slicer (Conduct Science, RWD‐BMCS‐00), and sections were immersed in 2% TTC (Sigma, T8877) in PBS for 5 min at 37°C. Sections were subsequently fixed in 4% PFA overnight at 4°C. Slices were scanned at 600 dpi resolution with a flatbed scanner (Scanjet G4050, Hewlett‐Packard). TTC stains healthy tissue red; thus, the absence of red stain was used to determine the lesion area. White lesion and total brain areas were traced and quantified in Fiji/ImageJ. The lesion areas in all slices were then added together to calculate the total lesion volume.

### 2.5 Brain Tissue Preparation and Immunohistochemistry (IHC)

Following euthanasia and perfusion, brains were harvested and incubated in 4% PFA overnight at 4°C, and then placed in 30% sucrose/PBS for at least 3 days. Brains were subsequently immersed in Tissue‐Plus O.C.T. Compound (Fisher HealthCare, 23‐730‐571) and frozen in isobutane surrounded by liquid nitrogen. Coronal sections (30 µm) were cut at −20°C using a Leica CM1950 cryostat, mounted onto gelatin‐coated positively charged slides (Globe Scientific, 1358 W), and dried overnight before storage at −80°C until immunostaining.

#### 2.5.1 Fluorescent IHC

Brain sections were rehydrated in PBS for 5 min prior to antigen retrieval via proteinase K treatment. In brief, tissues were treated with Proteinase K (Fisher Scientific, 20 µg/mL) in buffer (50 mM Tris Base, 1 mM EDTA, 0.5% Triton X‐100, pH 8.0) for 15–30 min at 37°C and washed in PBS twice for 5 min. Autofluorescence was blocked with Sudan Black B incubation (Fisher Scientific, BP109‐10, 0.1% in 70% EtOH, 10 min at RT), followed by rinsing in PBS until clear. Non‐specific antibody binding was blocked by incubating in 5% BSA/PBS for 45 min. Tissues were incubated in primary antibodies diluted in 5% BSA/PBS overnight at 4°C, then washed in PBS (four times, 15 min), and then incubated in species‐specific secondary antibodies diluted at 1:200 in 5% BSA/PBS for 1 h at RT. For fluorescent IHC, sections were washed in PBS (twice, 5 min), stained with DAPI (Thermo Fisher, 62247, 300 nM, 5 min), washed in PBS (twice, 5 min), and then rinsed with distilled H_2_O for 1 min. Sections were dried and mounted to coverslips in Aqua‐Poly/Mount (Polysciences, 18606).

For determination of C3, two different antibodies were used. The first, goat anti‐C3d (Bio‐Techne AF2655) was used for initial studies in 24 h, 3 months, and 9 months post‐MCAO samples. Later experiments for 7 days post‐MCAO samples employed a different antibody, rat anti‐C3 (all isoforms, Novus Biologicals NB200‐540), as later lots of the original C3d antibody were found to be no longer effective in immunohistochemistry.

#### 2.5.2 Colorimetric IHC

Tissues were treated similarly to fluorescent IHC, with the following alterations: After antigen retrieval, Sudan Black B incubation was omitted, and instead, non‐specific peroxidase activity was blocked by incubating sections in 0.01% H_2_O_2_/PBS for 5 min. Slides were rinsed in PBS (three times, 5 min), and Avidin/Biotin blocking was performed using the Avidin/Biotin Blocking Kit (Thermo Fisher, 004303) according to the manufacturer's instructions. Tissues were then blocked with 5% BSA/PBS (30 min, RT), incubated in primary antibody in 5% BSA/PBS overnight at 4°C. Sections were washed in PBS (five times, 15 min) and then incubated in biotinylated species‐specific secondary antibody (1:200, room temperature, 1 h). Slides were washed with PBS (five times, 5 min), and then incubated in VECTASTAIN Elite ABC‐HRP R.T.U Reagent (Vector Labs, PK‐7100) for 45 min, and washed in PBS (three times, 5 min). Tissues were then developed using the VECTASTAIN ImmPACT DAB Peroxidase Substrate Kit (Vector Labs, SK‐4105) for 3 min according to the manufacturer's instructions. All slides were developed for equal lengths of time. Slides were washed in PBS (three times, 5 min) and then in distilled H_2_O (1 min). After air‐drying overnight, slides were rinsed in Histo‐Clear (National Diagnostics, HS‐200) for 1 min and mounted with limonene mounting medium (Electron Microscopy Sciences, 17987‐01).

### 2.6 Image Acquisition and Analysis

Imaging was performed in the Optical Imaging Facility (supported by UTHSA and NIH‐NCI P30 CA54174). For each brain, 3–5 sections (30 µm thick) spaced 300 µm apart were imaged and analyzed by a blinded researcher. The unblinded results were pooled from *n* = 5–6 mice per group.

#### 2.6.1 Confocal Imaging and Analysis

Tissue sections with fluorescent staining were imaged on a Zeiss LSM710 laser scanning confocal microscope using a 20 × 0.8 NA (Plan‐Apo Chromat) objective. Tile‐scanned, *z*‐stacked (1 µm increments) images were acquired in the region of interest. Image analysis was performed by a blinded researcher using ImageJ containing the Fiji plugin suite.

To measure LRP1 expression in GFAP^+^ cells, the GFAP channel was used to create region of interest (ROI) selections to limit analysis to astrocytes: all color channels were converted to 8‐bit, images were despeckled to remove single‐pixel background, brightness of the GFAP channel was adjusted uniformly across samples for optimal visualization of astrocyte cell bodies and processes, and then the GFAP channel was thresholded to include only GFAP signal. A binary mask was created using the resultant thresholded GFAP image stack, pixels were inverted and dilated, allowing the red pixels in the GFAP channel to be created as a selection and added to the ROI manager. ROIs from the GFAP channel were used to select the same area in the 8‐bit LRP1 (green) channel, and this was performed in every slice of the z‐stack. The mean gray intensity and total area (mm^2^) in the selected ROIs were measured. Quantification of LRP1 expression in GFAP^+^ cells was performed by multiplying mean gray intensity and area. The resulting values for all Control samples were averaged, and results are expressed as a percentage of Control average.

To measure C3 expression in GFAP^+^ cells, the GFAP channel was used to create region of interest (ROI) selections to limit analysis to astrocytes: all color channels were converted to 8‐bit, images were despeckled to remove single pixels background, brightness of the GFAP channel was adjusted uniformly across samples for optimal visualization of astrocyte cell bodies and processes, and then the GFAP channel was thresholded to include only GFAP signal. A binary mask was created using the resultant GFAP stack, allowing the red pixels in the GFAP channel to be created as a selection and added to the ROI manager. ROIs from the GFAP channel were used to select the same area in the 8‐bit C3 (green) and DAPI (blue) channels, and this was performed in every slice of the z‐stack. The number of C3^+^ astrocytes and the total image area were measured. Quantification of C3 expression in GFAP^+^ cells was performed by dividing the number of C3^+^ astrocytes by area. Results are expressed as a percentage of Control‐Sham average.

For cell density analysis, the ROI area was measured, and the total number of positive cells was calculated using the FIJI/ImageJ cell counter within the ROI.

#### 2.6.2 Aperio Imaging

Tissue sections with colorimetric IHC staining and myelin staining were imaged on an Aperio VERSA 200 using a 20× brightfield objective. Images were analyzed by a blinded researcher using Fiji/ImageJ. For densitometry analysis to determine protein levels, images were converted to 8‐bit and inverted. Images were thresholded uniformly across samples to minimize background, and the mean intensity and %pixels above threshold were measured in the ROI. Labeling density was calculated as mean intensity multiplied by %pixels above threshold. Cell density analysis was performed as described above in Confocal Imaging and Analysis.

### 2.7 Statistical Analysis

All experiments and analyses were conducted by researchers blinded to the genotype and experimental condition. Respective sample sizes in each experiment are noted as appropriate. Males and females were used in all conditions. For image analysis, at least three sections spaced 300 µm apart were analyzed and pooled per mouse. In vitro analyses were performed with three biological replicates, with *n* = 3+ technical replicates per condition. Student's *t*‐test was used to compare two groups, and two‐way ANOVA with Tukey's HSD was used to compare more than two groups, with *α* = 0.05. Effect sizes are reported as partial eta squared (*ηp* ^2^) for ANOVA and eta squared (*η* ^2^) for *t*‐tests. GraphPad Prism software was used for all statistical analyses. Mean values with SEM are shown for the results. Additional materials and methods can be found within Supporting Information.

## 3 Results

### 3.1 Astrocyte‐Specific Knockout of LRP1 in Adult Mice

**FIGURE 1:**
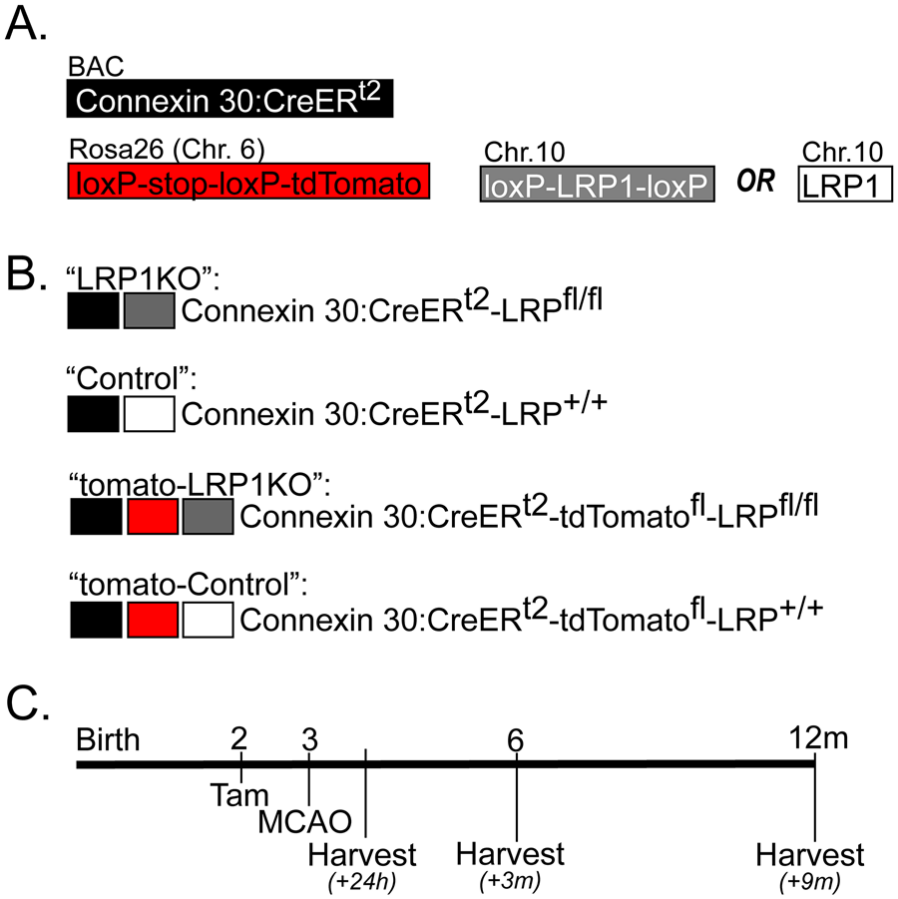
Conditional knockout of *Lrp1* in astrocytes of adult mice and experimental timeline. (A) Gene cassettes used for transgenic manipulation of *Lrp1* in adult mice. (B) LRP1KO mice were generated by crossing *Cx30‐CreER^T2^* mice (donated by F. Pfrieger, European Neuroscience Institute at Strasbourg, France) and *Lrp1^fl/fl^* mice (Jackson stock 012604). Cousins expressing *Cx30‐CreER^T2^* and wild‐type *Lrp1* (*Lrp1^+/+^*) were used for Control. Mice with tdTomato reporter were generated by crossing LRP1KO or Control mice with *stop^fl/fl^‐tdTomato* Ai14 reporter mice (Jackson stock 007914). (C) Timeline for tamoxifen injections, MCAO, and brain tissue harvesting.

To test a role for LRP1 in astrocyte function, we crossed *Cx30‐CreER^T2^* mice, *Lrp1^fl/fl^* mice, and *stop^fl/fl^‐tdTomato* mice to generate tamoxifen‐inducible astrocyte‐specific LRP1 knockout mice (*Cx30‐CreER^T2^, Lrp1^fl/fl^*; **LRP1KO**) as well as **tomato‐LRP1KO** (*Cx30‐CreER^T2^, stop^fl/fl^‐tdTomato, Lrp1^fl/fl^*) mice (Figure 1A,B). Cousins that express *Lrp1^+/+^* instead of *Lrp1^fl/fl^* were used for comparison (**Control** and **tomato‐Control**; Figure 1A,B). All mice received tamoxifen at 2 months of age. LRP1KO and Control mice received MCAO/sham surgeries 1 month later, then behavioral data and brain tissue were collected at 24 h, 3 months, and 9 months after surgery (Figure 1C). Western blotting of whole‐brain tissues revealed an approximate 50%–60% decrease in total LRP1 protein levels in the cortex, diencephalon, and cerebellum (Figure S1A,B). This reduction was further confirmed via immunohistochemistry (Figure S1C–F).

**FIGURE 2:**
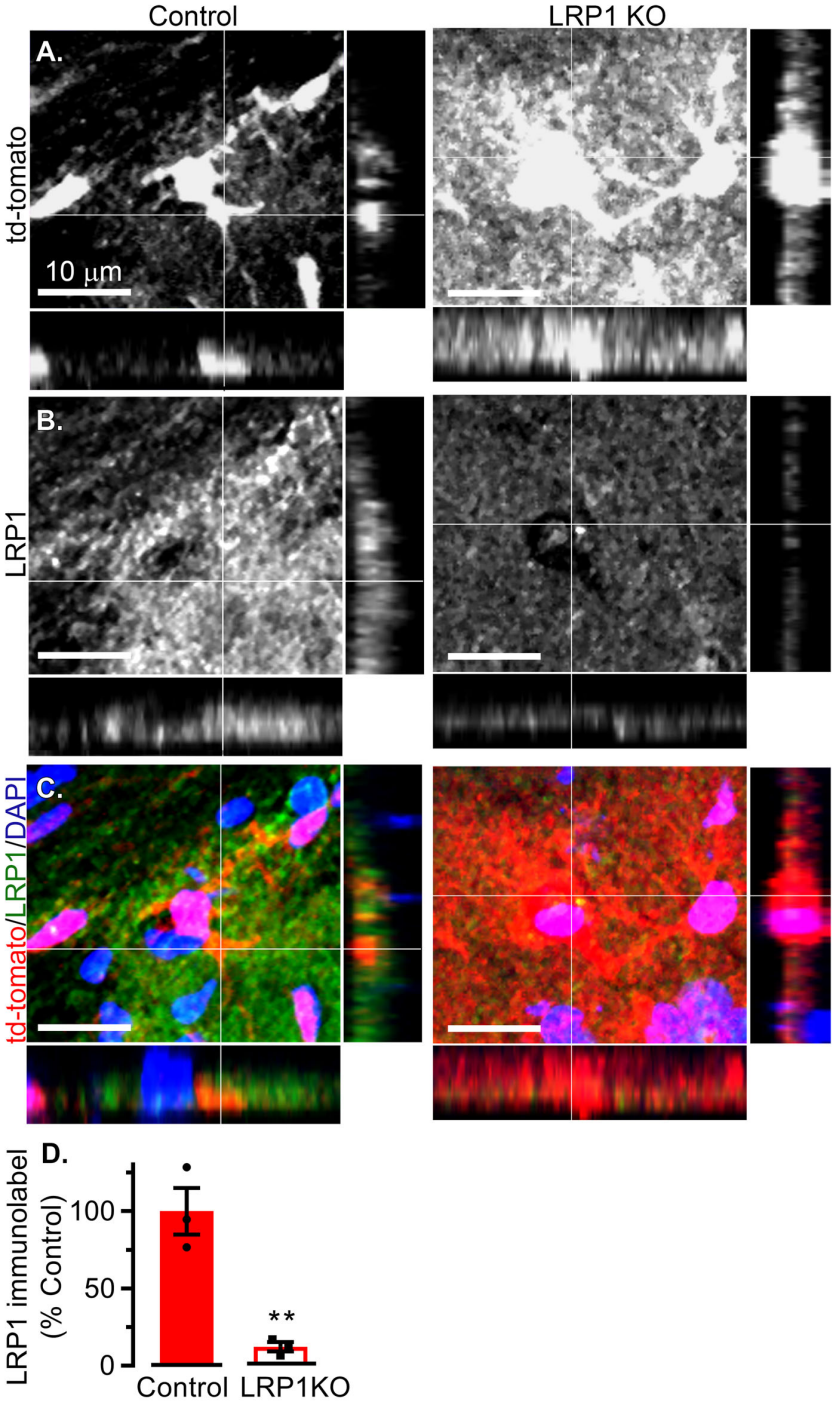
Astrocyte‐specific loss of LRP1 in *Cx30‐CreER^T2^* mice expressing the tdTomato reporter. Mice were harvested 1 month post‐tamoxifen, at 3 months of age. IHC was performed in tomato‐Control and tomato‐LRP1KO mice, showing signals of (A) tdTomato, (B) LRP1, and (C) tdTomato (red), LRP1 (green) and DAPI (blue) merged. (D) LRP1 immunolabeling was quantified in tdTomato^+^ astrocytes. Results are averages ± SEM pooled from 3–4 images/mouse in *n* = 3 mice/group. **p < 0.01 via Student's *t*‐test. Lack of asterisk indicates no significant differences.

Astrocyte‐specific loss of LRP1 was confirmed in tomato‐LRP1KO mice compared to tomato‐Control mice 1 month after tamoxifen treatment (Figure S2A). Primary astrocytes expressing tdTomato were isolated from both groups of mice via fluorescence‐activated cell sorting (FACS; Figure S2B,C). Western blotting of FACS‐sorted tdTomato^+^ astrocytes grown in cell culture confirmed a complete loss of LRP1 expression in tdTomato^+^ astrocytes of tomato‐LRP1KO mice (Figure S2D). Finally, colocalization of LRP1 with the tdTomato marker was confirmed via immunohistochemistry (Figure 2A‐‐C), revealing virtually no LRP1 signal in tdTomato^+^ cells from tomato‐LRP1KO mice (Figure 2D).

### 3.2 Astrocyte‐LRP1 Loss Exacerbates Motor Impairment at 24 h Post‐MCAO

**FIGURE 3:**
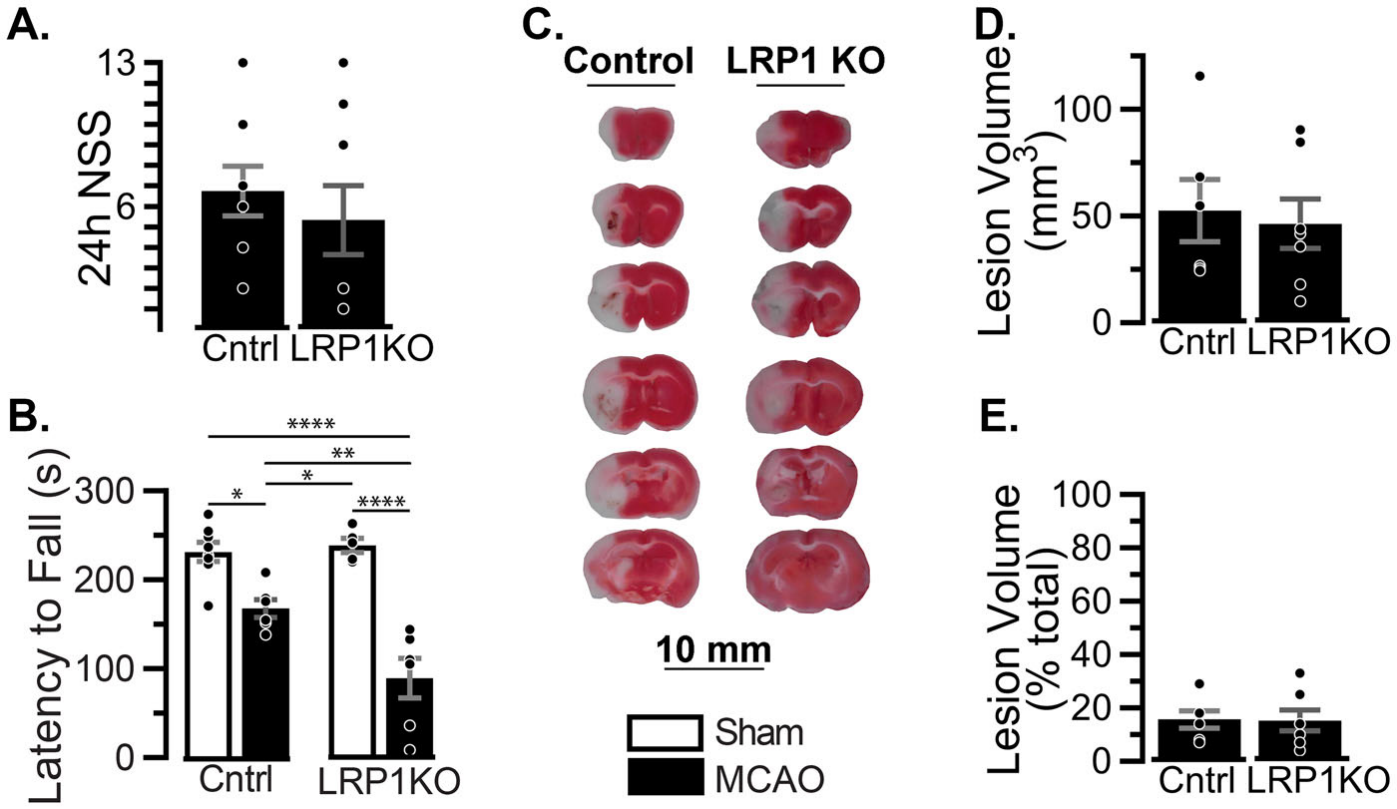
Acute recovery of LRP1KO mice 24 h after MCAO: Mice were subjected to MCAO at 3 months of age, 1 month after tamoxifen. Behavior and brain tissue were assessed 24 h later. (A) Neurological severity score of MCAO‐treated mice. (B) Motor coordination was measured via latency to fall from an accelerating rotarod, with effect sizes of genotype *ηp* ^2 ^= 0.227, surgery *ηp* ^2 ^= 0.727, and interaction *ηp* ^2 ^= 0.301. (C) Representative TTC‐stained brain sections of MCAO‐treated mice. Lesion volume was quantified (D) in mm^3^ and (E) as a percentage of the total brain volume. Results are averages ± SEM of *n* = 5–8 mice/group. Significant differences were tested via (A, D, and E) Student's *t*‐test or (B) two‐way ANOVA followed by Tukey's HSD. *p < 0.05; **p < 0.01; ****p < 0.0001. Lack of asterisk indicates no significant differences.

To investigate the impact of astrocyte‐LRP1 loss on post‐stroke recovery, LRP1KO and Control mice underwent transient middle cerebral artery occlusion (MCAO) 1 month after tamoxifen treatment (Figure 1C). Laser‐Doppler confirmed a 75% reduction in cortical blood flow compared to pre‐surgical values in both genotypes during MCAO (Figure S3). The filament was removed after 1 h and blood flow returned to normal (Figure S3). At 24 h post‐MCAO, neurological deficits were assessed using the modified neurological severity score (NSS). NSS scores did not differ significantly between genotypes, indicating comparable overall deficits at this time point (Figure 3A). However, accelerating rotarod results revealed significantly worse motor coordination in LRP1KO‐MCAO mice compared to Control‐MCAO (Figure 3B). We identified a very large main effect of surgery on rotarod performance (*ηp* ^2^ = 0.727), a large effect of genotype (*ηp* ^2^ = 0.227), and a large effect of the interaction between surgery and genotype (*ηp* ^2^ = 0.301), indicating that loss of astrocytic LRP1 exacerbated acute impairment in motor coordination induced by ischemia.

To determine if larger lesions contributed to the worsened motor impairment in LRP1KO‐MCAO mice, we stained brain tissue with TTC to identify necrotic tissue at 24 h post‐MCAO. We found no difference in lesion volume between LRP1KO and Control mice (Figure 3D,E). We further evaluated apoptosis via TUNEL staining (Figure S4A–D). While MCAO significantly increased apoptotic cells in both genotypes compared to sham, no difference was observed between LRP1KO and Control mice at 24 h post‐MCAO (Figure S4E).

### 3.3 Astrocyte‐LRP1 Loss Promotes Long‐Term Motor Function Recovery After MCAO

**FIGURE 4:**
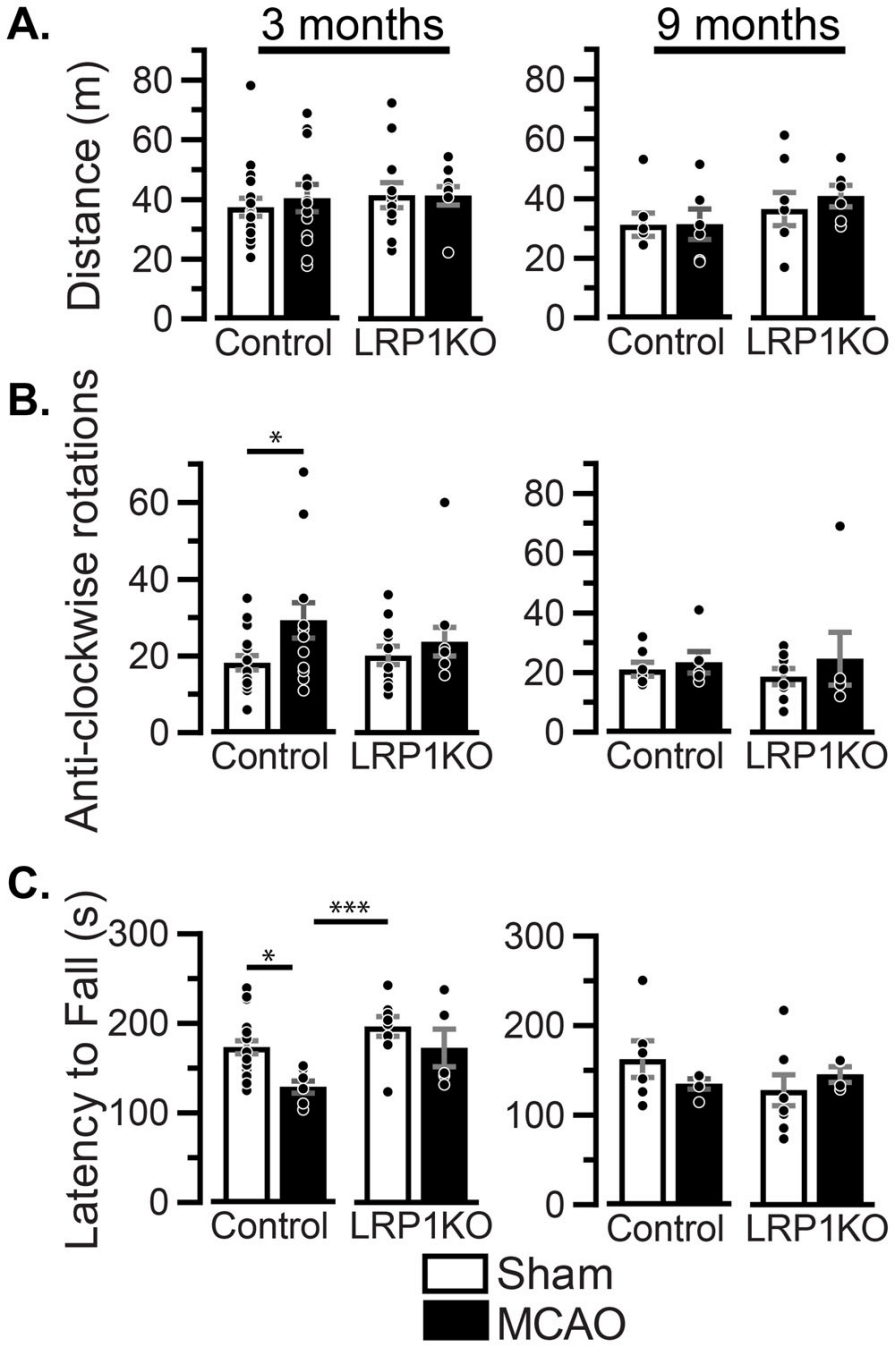
Long‐term functional recovery 3 and 9 months post‐MCAO: Mice were subjected to MCAO at 3 months of age, and behavior was measured 3 and 9 months later. (A) Distance travelled and (B) anti‐clockwise rotations in the open field, with surgery *ηp* ^2 ^= 0.094 and interaction *ηp* ^2 ^= 0.027 at 3 months post‐MCAO (*n* = 11–19 mice/group for 3 months post‐surgery, and *n* = 6–8 mice/group for 9 months post‐surgery). (C) Motor coordination was measured as latency to fall on the rotarod, with surgery *ηp* ^2 ^= 0.197, genotype *ηp* ^2 ^= 0.19, and interaction *ηp* ^2 ^= 0.022 at 3 months post‐MCAO (*n* = 5–19 mice/group for 3 months post‐surgery, and *n* = 4–8 mice/group for 9 months post‐surgery). Results are averages ± SEM, Comparisons were made within each time point for all experimental conditions via two‐way ANOVA followed by Tukey's HSD *p < 0.05 and ***p < 0.001. Lack of asterisk indicates no significant differences.

We next assessed long‐term recovery of LRP1KO and Control mice at 3 and 9 months after MCAO. Open field analysis revealed no significant differences in total distance traveled between genotypes or surgery treatment groups at either time point (Figure 4A). As expected, Control‐MCAO mice displayed an increase in anti‐clockwise rotations compared to Control‐sham mice at 3 months post‐surgery, suggesting a left‐side weakness (Figure 4B). This difference resolved in mice at 9 months post‐surgery (Figure 4B). In contrast, LRP1KO mice with MCAO did not exhibit increased anti‐clockwise rotations compared to sham at either time point (Figure 4B). Two‐way ANOVA identified a moderate‐to‐large main effect of surgery at 3 months (*ηp* ^2 ^= 0.094), a very small effect of genotype (*ηp* ^2 ^= 0.006), and a small‐to‐moderate effect of the genotype × surgery interaction (*ηp* ^2 ^= 0.027). Similarly, accelerating rotarod performance indicated worsened motor coordination in Control‐MCAO mice at 3 months post‐surgery compared to Control‐sham, as shown by reduced latency to fall. This difference between Control‐MCAO and Control‐sham was resolved by 9 months post‐MCAO (Figure 4C). On the other hand, LRP1KO‐MCAO mice did not exhibit reduced latency to fall at either 3 or 9 months after surgery compared to LRP1KO‐sham mice (Figure 4C). Two‐way ANOVA at 3 months post‐surgery revealed very large effects of both surgery (*ηp* ^2 ^= 0.197) and genotype (*ηp* ^2 ^= 0.19), with a small‐to‐moderate effect of the interaction between surgery and genotype (*ηp* ^2^ = 0.022), suggesting that astrocyte‐LRP1KO contributes to improved motor coordination after MCAO at this time point.

### 3.4 Astrocyte‐LRP1 Loss Does Not Reduce Apoptosis 3 and 9 Months After MCAO

To investigate if reduced cell death contributed to the improved motor function recovery observed in LRP1KO‐MCAO mice compared to Control‐MCAO at later time points, we evaluated cell death via TUNEL staining at 3 and 9 months post‐MCAO. While apoptosis was significantly elevated in all MCAO mice at 24 h post‐surgery, no significant differences in apoptotic cell numbers were detected between genotypes or surgical groups at 3 or 9 months (Figure S4). These findings suggest that the improved long‐term functional recovery in LRP1KO‐MCAO mice compared with Control‐MCAO is not attributable to an overall reduction in apoptotic cell death at these later time points.

### 3.5 Astrocyte‐LRP1 Knockout Does Not Alter Myelin Staining in the Chronic Phase Post‐Stroke

Recent research has discovered that astrocyte‐LRP1 is involved in the clearance of myelin debris (Clayton et al. 2024) after stroke and can contribute to secondary demyelination after stroke in mice (Fernandez‐Castaneda et al. 2013; Wan et al. 2022). To determine if the improved motor coordination at 3 months was associated with bulk alterations in myelin, tissues were stained with Black‐Gold II to label myelin. In both Control and LRP1KO mice, MCAO reduced the level of cortical and striatal stain within the right hemisphere compared to sham‐treated mice at 24 h after MCAO (Figure S5). No significant differences in myelin staining were observed at 3 and 9 months post‐surgery in the striatum or cortex, suggesting that the differences in motor coordination observed at 3 months post‐MCAO are not due to major disruption of myelination.

### 3.6 Astrocyte‐LRP1 Knockout Reduces Astrogliosis 3 Months After MCAO

**FIGURE 5:**
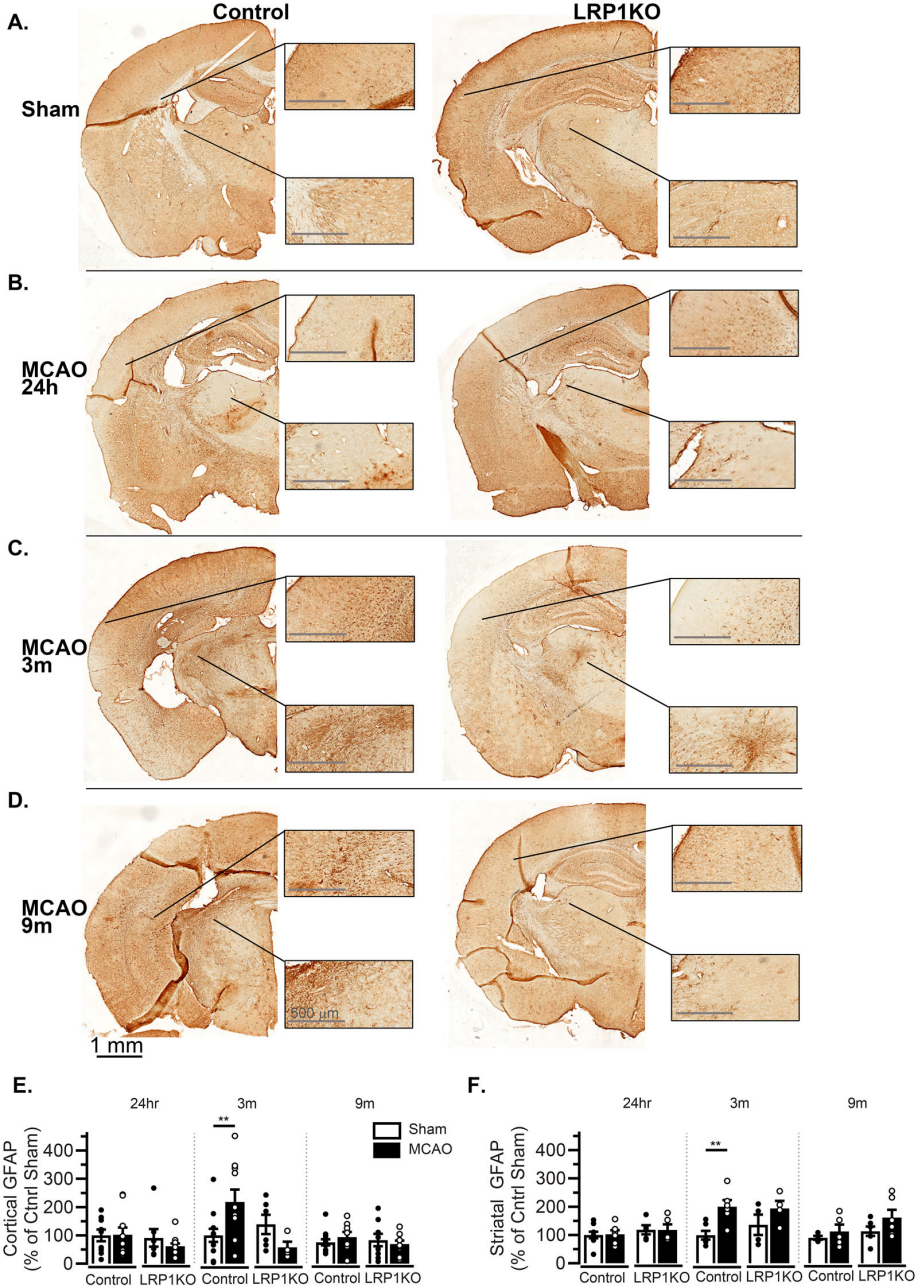
Astrogliosis after MCAO. Colorimetric GFAP immunolabeling was performed in harvested tissues. Representative images are shown for (A) sham surgery, (B) 24 h post‐MCAO, (C) 3 months post‐MCAO, and (D) 9 months post‐MCAO. Panels show enlarged views of the indicated regions, which were quantified in (E) cortex and (F) striatum as a percentage of GFAP levels in sham‐treated Control mice at that time point. Effect sizes (*ηp* ^2^) at 3 months post‐surgery are (E) surgery = 0.008, genotype = 0.08, interaction = 0.189, and (F) surgery = 0.427, genotype = 0.04, interaction = 0.068. Results are averages ± SEM pooled from 3–4 brain slices/mouse of *n* = 4–13 mice/group. **p < 0.01 via two‐way ANOVA followed by Tukey's HSD. Lack of asterisk indicates no significant differences.

Astrocyte reactivity, a hallmark of brain injury (Liddelow et al. 2017), was assessed via GFAP immunohistochemistry in the striatum and cortex at 24 h, 3 months, and 9 months post‐surgery (Figure 5A–F). No differences in astrogliosis were observed at 24 h post‐surgery (Figure 5B,E,F). However, at 3 months post‐surgery, Control‐MCAO mice displayed significantly increased astrogliosis compared to Control‐Sham mice, while LRP1KO‐MCAO mice did not exhibit significantly elevated astrogliosis compared to LRP1KO‐Sham mice (Figure 5C,E,F). Two‐way ANOVA indicated a moderate effect from the interaction between genotype and surgery in the cortex (*ηp* ^2 ^= 0.189), whereas in the striatum, differences in GFAP levels were primarily driven by surgery (*ηp* ^2 ^= 0.427). Any significant differences in astrogliosis were no longer observed at 9 months post‐MCAO, regardless of genotype and surgical condition (Figure 5D,E,F). To determine whether the reduced gliosis could be due to altered levels of astrocyte apoptosis at 24 h and 3 months post‐stroke, we measured the total percentage of GFAP^+^ TUNEL^+^ astrocytes in the striatal lesion (Figure S6A–H) and observed no differences in astrocyte‐specific apoptosis (Figure S6I).

### 3.7 Astrocyte‐LRP1 Knockout Does Not Affect Microglial Activation

Microglia play an integral role in neuroinflammation and stimulate astrogliosis (Liddelow et al. 2017). To determine if microglial activity could have contributed to the differential astrogliosis and function recovery observed, we assessed microglial density via Iba1 immunostaining in the striatal lesions. No significant differences were detected in Iba1^+^ cell density at 24 h post‐MCAO (Figure S7A–D,M). MCAO significantly increased Iba1^+^ cell density in the striatum at 3 and 9 months post‐surgery compared to sham‐treated animals; however, no significant differences were detected between LRP1KO and Control mice (Figure S7E–M). We next tested if LRP1KO affected neuroinflammatory microglial activation within lesions using CD68 immunolabeling (Simpson et al. 2007) (Figure S8). We found that CD68^+^ microglia numbers were consistent at 24 h post‐surgery (Figure S8A–D,M) but were increased 3 months after MCAO in both Control and LRP1KO mice compared to sham mice (Figure S8E–H,M). At 9 months post‐MCAO, CD68^+^ microglia were no longer increased in any groups (Figure S8I–L,M), suggesting microglial inflammation had resolved at this time point. Altogether, these findings suggest that astrocyte‐specific LRP1 loss does not significantly alter microglial activation following MCAO.

### 3.8 Astrocyte‐LRP1 Knockout Acutely Increases C3d Expression in Astrocytes

**FIGURE 6:**
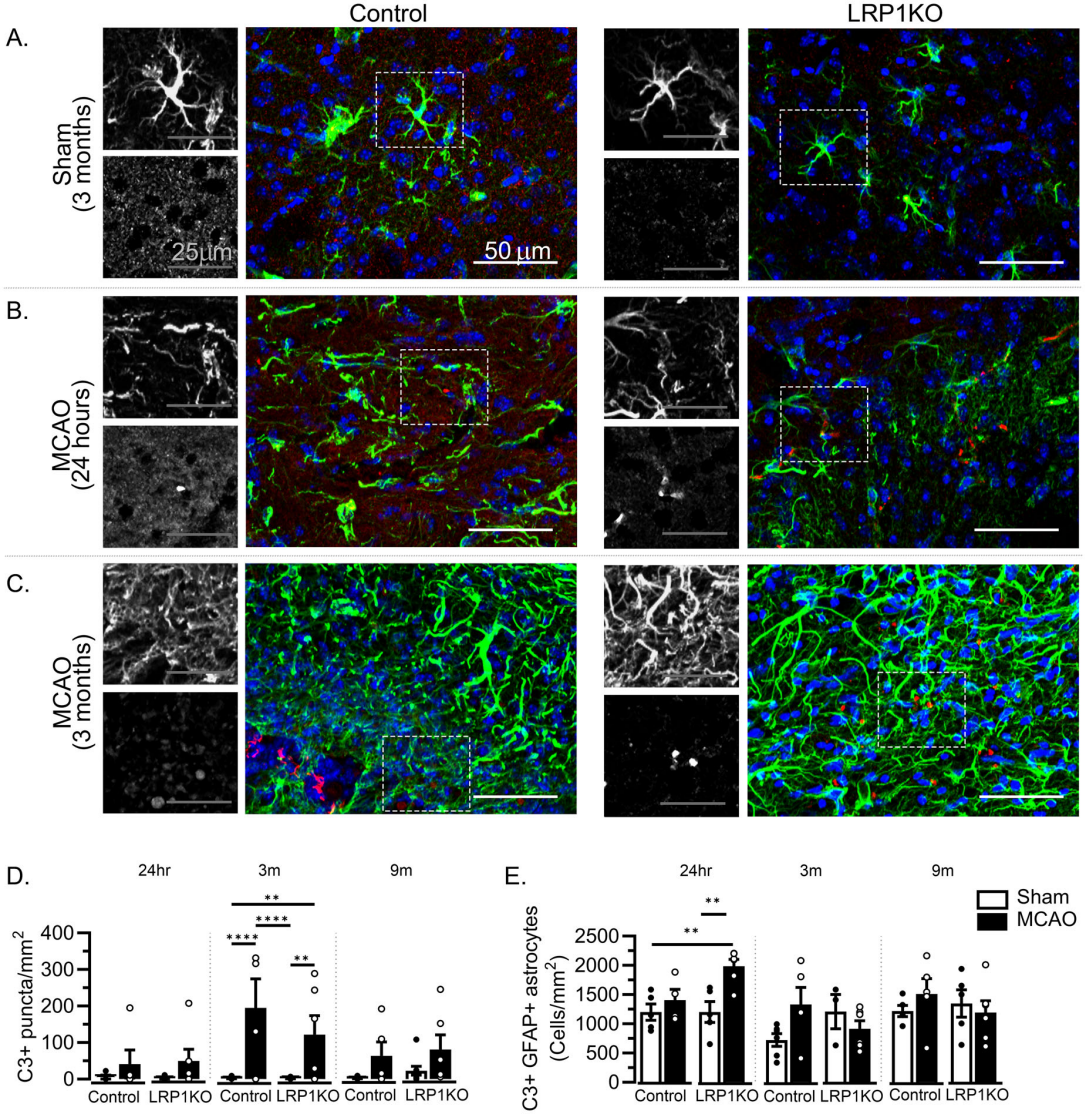
C3d expression after MCAO. Fluorescent immunolabeling of C3 and GFAP was performed, and representative images from within the striatum are shown for (A) sham‐treated mice at 3 months post‐surgery, (B) MCAO‐treated mice 24 h post‐surgery, and (C) MCAO‐treated mice 3 months post‐surgery. (D) Total C3^+^ puncta (*n* = 4–12 mice/group) and (E) C3^+^ GFAP^+^ astrocytes (*n* = 4–6 mice/group) were quantified. Effect sizes (ηp^2^) are (D) genotype = 0.053, surgery = 0.506, interaction = 0.054, and (E) genotype = 0.186, surgery = 0.395, interaction = 0.184. Results are expressed as averages ± SEM pooled from 3–4 brain slices/mouse. *p < 0.05, **p < 0.01, ***p < 0.001, ****p < 0.0001 via two‐way ANOVA followed by Tukey's HSD. Lack of asterisk indicates no significant differences.

Activation of the immune response can be approximated by measuring the expression of complement pathway component C3, more specifically C3d, a cleaved portion of the full‐length C3 (Chen et al. 2022), much of which is produced by astrocytes in the CNS (Veerhuis et al. 2011). First, we measured the total number of C3d^+^ puncta (whether associated with astrocytes or not) within the striatum (Figure 6) and found that MCAO increased the total number of C3d^+^ puncta to the same extent at 3 months post‐surgery in both Control and LRP1KO mice (Figure 6A,C,D). This increased total C3d expression was not observable either at 24 h or 9 months post‐stroke. We further counted the total number of astrocytes co‐expressing GFAP and C3d, as astrocyte‐associated C3 has been implicated as a marker for inflammatory, potentially neurotoxic‐type reactive astrocytes (Liddelow et al. 2017). We found that 24 h after MCAO, LRP1KO mice had significantly increased numbers of C3d^+^ GFAP^+^ astrocytes compared to sham‐treated Control and LRP1KO mice (Figure 6B,E), which was not apparent at 3 and 9 months after MCAO (Figure 6E). Effect sizes indicated that at 24 h post‐MCAO, surgery accounted for the majority of variance in astrocyte‐associated C3d expression, with smaller contributions from genotype and the genotype × surgery interaction.

### 3.9 Mice With Astrocyte‐Specific Loss of LRP1 Have Reduced Cell Death at 7 Days Post‐MCAO

**FIGURE 7:**
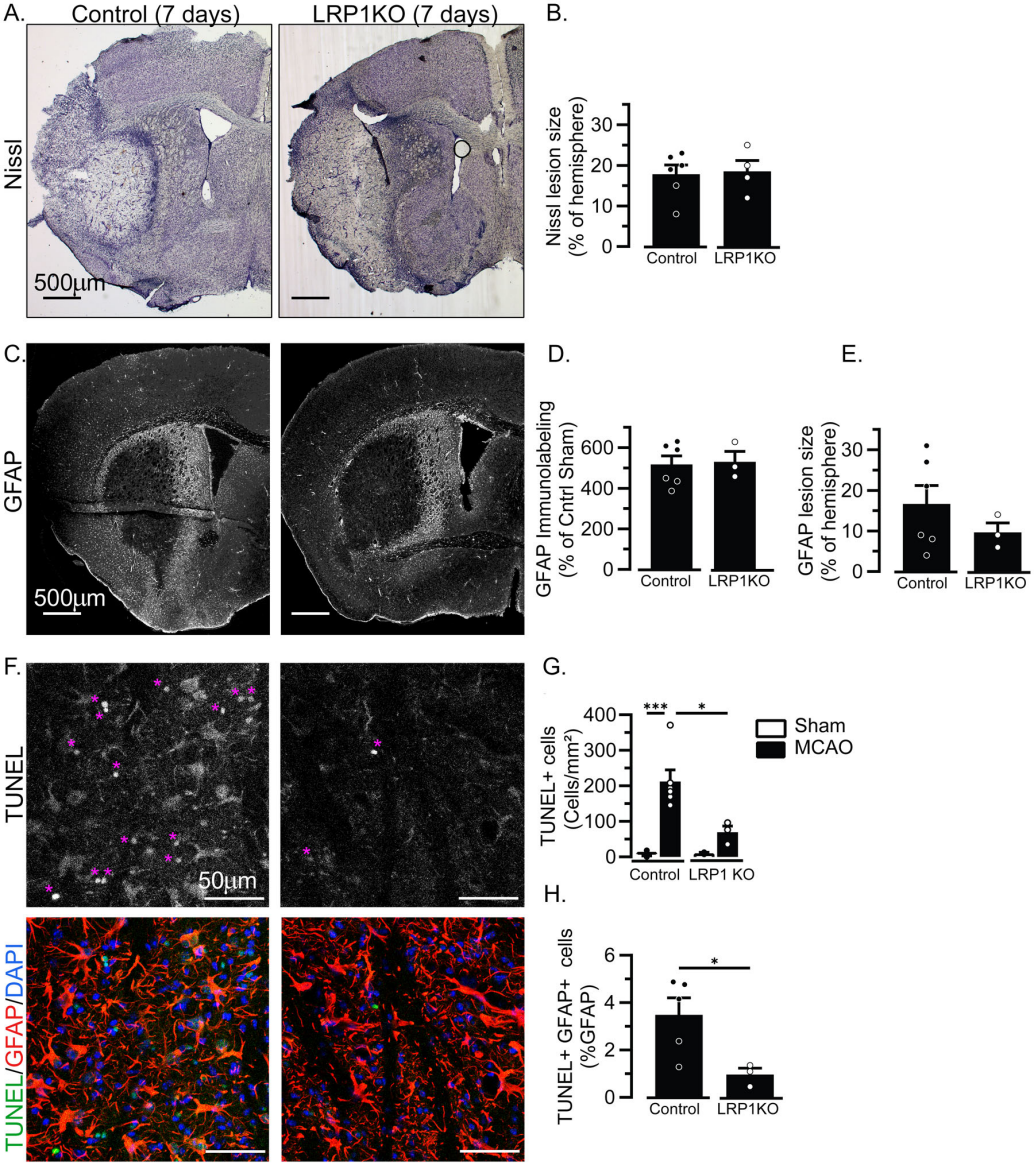
Recovery of LRP1KO mice 7 days after MCAO: Mice were subjected to MCAO at 3 months of age, 1 month after tamoxifen. Brain tissue was assessed 7 days later. (A) Representative Nissl‐stained tissue sections from Control and LRP1KO mice. (B) Quantification of lesions from Nissl‐stained tissue. Results are averages ± SEM pooled from 3–5 images/mouse in n = 4‐6 mice/group. (C) Representative fluorescent images of GFAP‐labeled tissues in Control and LRP1KO mice. (D) Quantification of GFAP immunolabeling on the entire ipsilateral hemisphere was determined by multiplying the intensity of fluorescent signal by percentage of pixels above a uniform thresholded value, expressed as a percentage of sham‐treated Control mice. Results are averages ± SEM pooled from 3–6 images/mouse in *n* = 3–6 mice/group. (E) The area of the lesion encircled by GFAP^+^ gliosis was measured and is expressed as a percentage of the ipsilateral hemisphere. Results are averages ± SEM pooled from 3–6 images/mouse in *n* = 3–6 mice/group. (F) Representative fluorescent images of tissue sections subjected to TUNEL and GFAP immunolabeling. Images were acquired in the dorsal penumbra within the striatum, in between the lateral ventricle and the lesion edge. The top panels represent TUNEL signal only, and the bottom panels represent the merged image with TUNEL in green, GFAP in red, and DAPI^+^ nuclei in blue. Pink asterisks label TUNEL^+^ nuclei. (G) The total number of TUNEL^+^ cells. Results are averages ± SEM pooled from 3–6 images/mouse in *n* = 3–6 mice/group. *p < 0.05, **p < 0.01 via two‐way ANOVA followed by Tukey's HSD. (H) The total number of TUNEL^+^ GFAP^+^ astrocytes expressed as a percentage of total number of GFAP^+^ astrocytes. Results are averages ± SEM pooled from 3–6 images/mouse in *n* = 3–6 mice/group. *p < 0.05 via Student's *t*‐test. Lack of asterisk indicates no significant differences.

Our data suggest that outside of the acute ischemic phase of 24 h, LRP1KO mice experienced accelerated recovery. To better resolve the acute timeline of recovery, the effect of astrocyte‐specific loss of LRP1 was measured near the end of the acute recovery phase, at 7 days post‐MCAO (Figure 7). The average NSS score was not significantly different for Control mice or LRP1KO mice at 7 days post‐MCAO (5.2 ± 2.3, *n* = 6 vs. 3.8 ± 2.2, *n* = 4), indicating mice were similarly impaired at this time point. Lesion size was measured on Nissl‐stained tissue sections, and no differences were observed in Control or LRP1KO mice (Figure 7A,B). Gliosis was also measured by immunolabeling for the astrocyte marker GFAP (Figure 7C), and no significant differences in either GFAP intensity or size of the gliotic scar were observed (Figure 7D,E). Astrocyte‐associated C3 expression was also measured, this time with an antibody directed to the full‐length C3 that measures multiple isoforms of C3. At this time point, we could not detect significant differences in full‐length C3 expression, although the majority of C3 was astrocyte‐associated (Figure S9A,B). Finally, we measured the amount of cell‐death within the penumbra of the ischemic injury via TUNEL labeling (Figure 7F). In Control mice subjected to MCAO, we observed significantly increased density of TUNEL^+^ cells compared to sham‐operated mice. However, the density of TUNEL^+^ cells was not significantly increased in LRP1KO mice subjected to MCAO. In fact, the density of TUNEL^+^ cells was significantly reduced in LRP1KO‐MCAO mice compared to Control‐MCAO mice (Figure 7G). We also counted the number of TUNEL^+^ astrocytes as a percentage of the GFAP^+^ astrocyte population and similarly observed reduced astrocyte cell death in LRP1KO mice compared to Control mice 7 days after MCAO (Figure 7H).

## 4 Discussion

Overall, we present our findings, which suggest that loss of astrocyte LRP1 in adult mice can affect outcomes after ischemic stroke. In the acute phase, we did not observe any significant differences in lesion size at 24 h or 7 days after stroke. However, at 24 h post‐stroke, LRP1KO mice had exacerbated motor impairment (Figure 3). Such results are potentially surprising, as we would have expected that motor impairments would be driven predominantly by lesion size differences. We did not further interrogate the mechanism underlying our finding. However, multiple mechanisms could contribute to worsened behavioral recovery despite similar lesion sizes, including non‐lesion disruption of synaptic function (which might be caused by neuroinflammation or direct astrocyte modulation of synaptic function), or even decreased network compensatory response to loss of circuitry. Further study is required.

At this same 24 h post‐stroke time point, we discovered that LRP1KO mice with MCAO had a greater density of C3‐expressing GFAP^+^ astrocytes within the striatal lesion compared to LRP1KO‐Sham mice (Figure 6). Other studies have described C3 as a potential marker for astrocytes that have acquired maladaptive, neurotoxic functions, failing to support neuronal health after injury (Liddelow et al. 2017; Renz et al. 2024). A recent study by Zhou et al. similarly discovered that knockdown of LRP1 impairs the ability of astrocytes to transfer mitochondria to neurons after ischemia as an acute protective mechanism, causing increased ischemic damage (Zhou et al. 2024). Taken together, these results suggest that the loss of LRP1 function in astrocytes could impair acute protective processes. Whether LRP1 influences acute astrocyte reactive transformation after damage, and whether this is a cause of worsened acute condition after ischemia, will require further investigation using additional studies, including potentially electrophysiological studies, refined analyses of astrocyte function, and potentially additional behavioral tests.

While our understanding of the influence of LRP1 on astrocyte functionality is limited, Liu et al. have found that loss of astrocyte LRP1 can exacerbate pathological accumulation of amyloid β (Aβ), and Romeo et al. found that in vitro deletion of LRP1 from astrocytes altered neuronal synaptogenesis and activity (Romeo et al. 2020). Therefore, astrocyte LRP1 may be involved in ongoing, long‐term functional regulation of processes which are beneficial to neuronal function, for instance by clearing amyloid β, which can accumulate as a result of ischemic injury (Pluta et al. 2021) and contribute to vascular cognitive impairment and dementia (Van Nostrand 2016). Given this, we were surprised to observe that astrocyte‐LRP1KO appears to promote long‐term recovery after stroke. Indeed, the first signs of this are observable by 7 days after ischemic injury. Here, our mouse model shows signs of expedited recovery, most notably by a lack of significantly greater numbers of TUNEL^+^ cells within the penumbra of LRP1KO mice.

Importantly, LRP1KO and Control mice exhibited similar lesion sizes and apoptosis levels acutely after stroke, suggesting that the observed differences in behavioral outcomes were not due to variations in stroke severity. Instead, we observe improved motor coordination, reduced circling behavior, and reduced astrogliosis near the lesion at 3 months post‐stroke. Potential explanations for this reduced astrogliosis require further investigation. Our results suggest that microglial activation remained consistent between Control and LRP1KO mice (Figures S7 and S8), therefore reducing the likelihood that microglia‐involved inflammatory pathways were driving the observed differences in astrogliosis. Instead, it appears that differential astrocyte reactivity may underlie the accelerated behavioral recovery by 3 months post‐stroke. At this time point, we observed that C3^+^ astrocytes are similar between groups, suggesting that the acute upregulation of this subtype of astrocytes has resolved. Whether that resolution is due to increased death of potentially maladaptive astrocytes, or accelerated reversion to homeostatic astrocytes, or some other explanation, would require investigation at time points between 7 days and 3 months after ischemia. Our data suggest that the total number of astrocytes is consistent between LRP1KO and Control mice at 7 days post‐MCAO, but that the total number of TUNEL^+^ astrocytes has decreased. This could suggest potential reversion of phenotype, but it would require more careful interrogation at intervening time points. It is also important to note that other, non‐astrocyte‐driven effects could underlie our observations. For example, myelination can differentially affect neural function. In the chronic phase post‐stroke, our initial measurement of myelin staining showed no overt differences; thus, we did not investigate further whether more subtle changes in myelin or myelin function occurred. However, from this study, we cannot rule out the possibility of alterations in myelination or function in LRP1KO mice.

In summary, our findings reveal a complex role for astrocyte‐LRP1 in post‐stroke recovery. Our study has some limitations. It fails to investigate the effects of astrocyte‐LRP1KO more thoroughly in the subacute phase, from 3 days to 1 month after stroke, but our findings suggest that dynamic processes may be at play during this phase, which accelerates recovery. Additionally, the Connexin‐30‐driven expression of Cre has previously been shown to cause only about 20%−40% of astrocytes to express Cre within the cortex, hippocampus, and striatum (Slezak et al. 2007). Given this, it is not clear if the observed phenotype is due to a compensatory response of wild‐type astrocytes or intrinsic effects of the LRP1KO astrocytes. Nevertheless, our study does suggest that in the chronic phase after stroke, improved recovery may be possible through the targeting of astrocyte‐LRP1 or its downstream effectors. This is especially significant given that stroke is currently the primary cause of long‐term disability in the United States and a significant risk factor for the development of dementia and neurodegenerative disease (Tsao et al. 2023). Further studies are warranted to elucidate the precise mechanisms by which astrocyte‐LRP1 regulates astrocyte reactivity and positively contributes to long‐term stroke outcomes.

## Author Contributions

M.W., S.N.A., and N.L.S. were responsible for behavioral analysis, histological analysis, data analysis, and presentation. P.R. managed the colony and provided technical assistance. S.S. performed rodent surgeries and provided technical assistance. All authors contributed to experimental design, manuscript writing, and editing.

## Funding

This study was supported by AHA 15BGIA2509029, VA CDA 1K2BX003240, and NINDS R01NS132778 to N.L.S. Support to M.W. was provided by T32AG082661, NIH T32GM113896, and T32GM145432. Support to S.N.A. was provided by TL1‐TR002647.

## Conflicts of Interest

The authors declare no conflicts of interest.

## Supporting information




**Supporting Materials**: brb371300‐sup‐0001‐SuppMat.pdf

## Data Availability

All data is available in raw and edited form upon request to the corresponding author.
