# Supplementary material for: Knockout of Low‐Density Lipoprotein Receptor‐Related Protein 1 From Astrocytes in Adult Mice Accelerates Long‐Term Functional Recovery After Ischemic Stroke
Source: Brain Behav. 2026 Mar 31;16(4):e71300. doi: 10.1002/brb3.71300 (PMC13112003; doi:10.1002/brb3.71300)
Supplement: Supplementary file 1 — Supporting Materials: brb371300‐sup‐0001‐SuppMat.pdf [file BRB3-16-e71300-s001.pdf]

Supplemental Information

Materials and Methods:

Terminal deoxynucleotidyl transferase-mediated dUTP nick end labeling (TUNEL) labeling:

The DeadEnd Colorimetric TUNEL System (Promega, G7130) or Fluorometric TUNEL System (Promega, G3250) were used to label fragmented nuclear DNA in apoptotic cells according to manufacturer's instructions. For colorimetric images, tissues were counterstained with hematoxylin for 30 seconds, rinsed in distilled H_2O until clear, and then rinsed quickly in 0.3% HCl/70% EtOH. After rinsing again in distilled H_2O, tissues were air-dried overnight. The next day, tissues were rinsed in Histo-Clear (National Diagnostics, HS-200) for 1 min and mounted in limonene mounting medium (Electron Microscopy Sciences, 17987-01). For fluorometric images, tissue was counterstained with DAPI (300 nM, 5 min) prior to mounting in Aqua-Poly/Mount.

Myelin Staining:

The Black-Gold II Myelin Ready-to-Dilute Staining Kit with Toluidine Blue O Counter Stain (Biosensis, TR-100-BG) was used according to manufacturer's instructions to label myelinated axons and counterstain Nissl bodies and nuclei. Slides were then mounted with Permount (Fisher Scientific, SP15-500). Myelination was measured in the striatum and cortex on the right side of brain tissue, and expressed as a percentage of the Control-sham mean at each timepoint.

Western blotting:

LRP1 levels across brain regions were assessed 2 weeks after tamoxifen treatment in Control and LRP1KO mice (n=6 mice/group). Tissues or cellular pellets were homogenized in 1mL cell lysis buffer (50 mM Tris, 150 mM NaCl, 1% Triton X-100, cOmplete Mini Protease Inhibitor Cocktail (Millipore Sigma, 11836153001) and PhosSTOP Phosphatase Inhibitor (Millipore Sigma, 04906845001) and sonicated 3 times for 10 seconds at 30%. After centrifugation (4°C, 16400 rpm, 10min), the pellet was discarded and supernatant was used for western blotting after determination of protein concentration via BCA assay (Thermo Scientific, 23227). Twenty µg total protein from each sample was subjected to SDS-PAGE and transferred onto a nitrocellulose membrane (LI-COR, 926-31092). Membrane was then blocked with Intercept Protein-Free Blocking Buffer (LI-COR, 927-90001) for 1 hr at room temperature before incubating in primary antibody (diluted in blocking buffer) overnight at 4°C. The next day, the membrane was washed in 1% Tween 20/PBS (4X, 10 min) and then incubated in secondary antibody (diluted in blocking buffer) at room temperature for 1 hr. Membrane was washed twice in 1% Tween 20/PBS for 10 min and once in PBS for 10 min. The membrane was visualized on the Li-Cor Odyssey DLx Imager. Expression was measured in FIJI/ImageJ by measuring densitometry of protein bands, and results are expressed as a percentage of control.

Primary antibody used was polyclonal rabbit anti-LRP1 custom generated by Abclonal via immunization of the Keyhole-Limpet Hemocyanin conjugated LRP1 peptide corresponding to the 13 carboxy terminal amino acids (GRGPEDEIGDPLA) of LRP1 (NM_002332) into rabbits, similar to previously published studies.[22]

Astrocyte isolation and culture:

Cells were triturated from whole brains of tomato-LRP1KO and tomato-control mice 1 month after tamoxifen (n=3 mice/genotype). Papain (120U, Worthington Biochemical Corporation, Lakewood, NJ) was prepared in 5 mL of DMEM/F12 (Sigma-Aldrich, D8062) and incubated at 37˚C for 30 min to activate the enzyme, and then DNAase (4 mg/ml) was added to the papain/media, and the mixture was filtered through a 0.45 µm filter (Pall Life Sciences, New York place). Mice were humanely euthanized with isoflurane (5% in Oxygen) and the whole brain was harvested and rinsed in ice cold HIB buffer (30 mM KCl, 5 mM NaOH, 5 mM $NaH_2PO_4H_2O$, 0.5 mM $MgCl_2.6H_2O$, 21 mM Na Pyruvate, 5.5 mM D-glucose, 200 mM D-Sorbitol at pH 7.5, filter sterilized). Brain was minced in HIB using a sterile razor blade and transferred to a conical tube. Filtered media was added to brains and incubated for 10 min at 37˚C with gentle agitation. Homogenate was triturated (10 times) with a 10 mL pipette and then centrifuged (10 min, 400 xg, 4˚C). The pellet was re-suspended in 22% Percoll (P4937 Sigma Aldrich, St. Louis, MO) in DMEM/F12. Homogenate was centrifuged (10 min, 400 xg, 4˚C), and the myelin-containing top fraction was removed. The bottom layer was washed 1X in HIB, and after centrifugation (10 min, 400 xg, 4˚C), the pellet was triturated in HIB again (10 times) with 10 mL pipette fitted with a 200µL pipetting tip (Vertex, Lodi, California). Homogenate was filtered using a 70µm cell strainer (BD Falcon, Franklin Lakes, NJ) and centrifuged (10 min, 400 xg, 4˚C), and the pellet was re-suspended in HIB (1 mL). Cells were sorted for tdTomato at the UT Health SA Flow Cytometry Core Facility, which is supported by UT Health, NIH-NCI P30 CA054174-20 (CTRC at UT Health) and UL1 TR001120 (CTSA grant). The sorted tdTomato+ astrocytes were grown in Astromedia:

DMEM/F12 (Sigma-Aldrich, D8062), 10% fetal bovine serum (Sigma-Aldrich, F2442), 1X Primocin (Invivogen, ant-pm-05).

Major Resources Table

Animals (in vivo studies)

Species	Vendor or Source	Background Strain	Sex	Persistent ID / URL
Connexin 30-CreERT2	Frank Pfrieger, European Neuroscience Institute, Strasbourg, France	C57bl/6	M/F	
LRP1 fl/fl	Jackson Laboratories	C57bl/6	M/F	B6;129S7-Lrp1^{tm2Her}/J<br>Jackson stock 012604
tdTomato Ai14	Jackson Laboratories	C57bl/6	M/F	B6.Cg-Gt(ROSA)26Sortm14(CAG-tdTomato)Hze/J<br>Jackson stock 007914

Primary Antibodies

Antibody	Vendor or Source	Catalog #	Working concentration	Lot # (preferred but not required)	Persistent ID / URL
Goat anti-C3	Bio-Techne	AF2655	1:100		RRID: AB_2066622
Rat anti-C3 11H9	Novus Biologicals	NB-200-540	1:100		RRID:AB_535612
Rabbit anti-CD68	Abcam	AB125212	1:200		RRID: AB_10975465
Chicken anti-Glial Fibrillary Acidic Protein	EMD Millipore	AB5541	1:500		RRID: AB_177521
Goat anti-IBA1	Abcam	AB5076	1:200		RRID: AB_2224402
Rabbit anti-LRP1 (IHC)	Abcam	AB92544	1:100		RRID: AB_2234877
Rabbit anti-LRP1 (WB)	Abclonal, custom-generated polyclonal antibody	Project AP19360, E8060	1:500		

Secondary Antibodies

Antibody	Vendor or Source	Catalog #	Working concentration	Lot # (preferred but not required)	Persistent ID / URL
Donkey anti-chicken Alexa 488	Jackson	703-545-155	1:200		RRID: AB_2340375
Donkey anti-chicken Alexa 594	Jackson	703-585-155	1:200		RRID: AB_2340377
Donkey anti-chicken biotinylated	Thermo Fisher	SA1-72003	1:200		RRID: AB_923385
Donkey anti-goat Alexa 568	Invitrogen	A-11057	1:200		RRID: AB_2534104
Donkey anti-goat biotinylated	Abcam	AB6884	1:200		RRID: AB_954842
Donkey anti-rabbit Alexa 488	Invitrogen	A-21206	1:200		RRID: AB_2535792
Donkey anti-rabbit biotinylated	Thermo Fisher	31821	1:200		RRID: AB_228212

Donkey anti-rabbit IRDye 800	Li-COR	925-32213	1:25,000		RRID: AB_2715510
Donkey anti-rat Alexa 488	Invitrogen	A21208	1:200		RRID:AB_2535794

Other

Description	Source / Repository	Persistent ID / URL
2, 3,5, triphenyltetrazolium chloride (TTC)	Sigma	T8877
Proteinase K	Fisher Scientific	50-213-634
Sudan Black B	Fisher Scientific	BP109-10
Hydrogen peroxide (H_2O_2), 35% w/w	Thermo Fisher	L14000.AP
Bovine Serum Albumin	Sigma	A3294
Avidin/Biotin Blocking Kit	Thermo Fisher	004303
VECTASTAIN® Elite® ABC-HRP Reagent, Peroxidase, R.T.U.	Vector Labs	PK-7100
ImmPACT® DAB Substrate Kit, Peroxidase (HRP)	Vector Labs	SK-4105
DeadEnd™ Colorimetric TUNEL System	Promega	G7130
DeadEnd™ Fluorometric TUNEL System	Promega	G3250
4',6-diamidino-2-phenylindole (DAPI)	Thermo Fisher	62247
Black-Gold II Myelin Ready-to-Dilute Staining Kit with Toluidine Blue O Counter Stain	Biosensis	TR-100-BG
Histo-Clear	National Diagnostics	HS-200
Aqua-Poly/Mount	Polysciences	18606
Limonene-Mount	Electron Microscopy Sciences	17987-01
Permount	Fisher Scientific	SP15-500
Tissue-Plus™ O.C.T. Compound	Fisher HealthCare	23-730-571
Diamond® White Glass Charged Slides	Globe Scientific	1358W
Pierce™ BCA Protein Assay Kit	Thermo Scientific	23227
cOmplete™, Mini Protease Inhibitor Cocktail	Millipore Sigma	11836153001
PhosSTOP™	Millipore Sigma	04906845001
Odyssey® Nitrocellulose Membranes	LI-COR	926-31092
Intercept® (PBS) Protein-Free Blocking Buffer	LI-COR	927-90001
Dulbecco's Modified Eagle's Medium/Nutrient Mixture F-12 Ham	Sigma-Aldrich	D8062
Fetal Bovine Serum	Sigma-Aldrich	F2442
Primocin®	Invivogen	ant-pm-05

ARRIVE GUIDELINES

The ARRIVE guidelines (https://arriveguidelines.org/) are a checklist of recommendations to improve the reporting of research involving animals. Key elements of the study design should be included below to better enable readers to scrutinize the research adequately, evaluate its methodological rigor, and reproduce the methods or findings.

Study Design

Groups	Sex	Age	Number (prior to experiment)	Number (after termination)	Littermates (Yes/No)	Other description
Control-sham	M/F	3-12 m/o	30	26	No	Cousins to LRP1KO mice
Control-MCAO	M/F	3-12 m/o	45	24	No	
LRP1KO-sham	M/F	3-12 m/o	29	24	No	Cousins to Control mice
LRP1KO-MCAO	M/F	3-12 m/o	45	25	No	

To reduce numbers of mice bred for experimentation, homozygous littermates were bred to generate experimental mice. Thus, LRP1KO and Control mice are one generation removed from each other.

Sample Size: Please explain how the sample size was decided. Please provide details of any a *prior* sample size calculation, if done.

Previous power analyses and experiments in the MCAO models have shown that at least 6 animals are necessary to obtain statistical significance below 0.05 for the detection of a 30% difference in brain damage between groups. For behavioral determination, at least 10 animals are needed to obtain the same power. In our hands, up to 15 mice are needed for surgery and long-term experiments, because MCAO surgery and the long time course cause some attrition of mice by the end point.

Inclusion Criteria

none

Exclusion Criteria

Mice must be able to remain on the rotarod moving at 20 RPM for 100 seconds. This test is conducted after rotarod training, the week before surgery.

Randomization

Mice were randomly divided into surgical groups as they were born.

Blinding

Experimenters performing behavioral studies and tissue analysis were blinded to experimental genotype and manipulation.

Supplemental Figure 1:

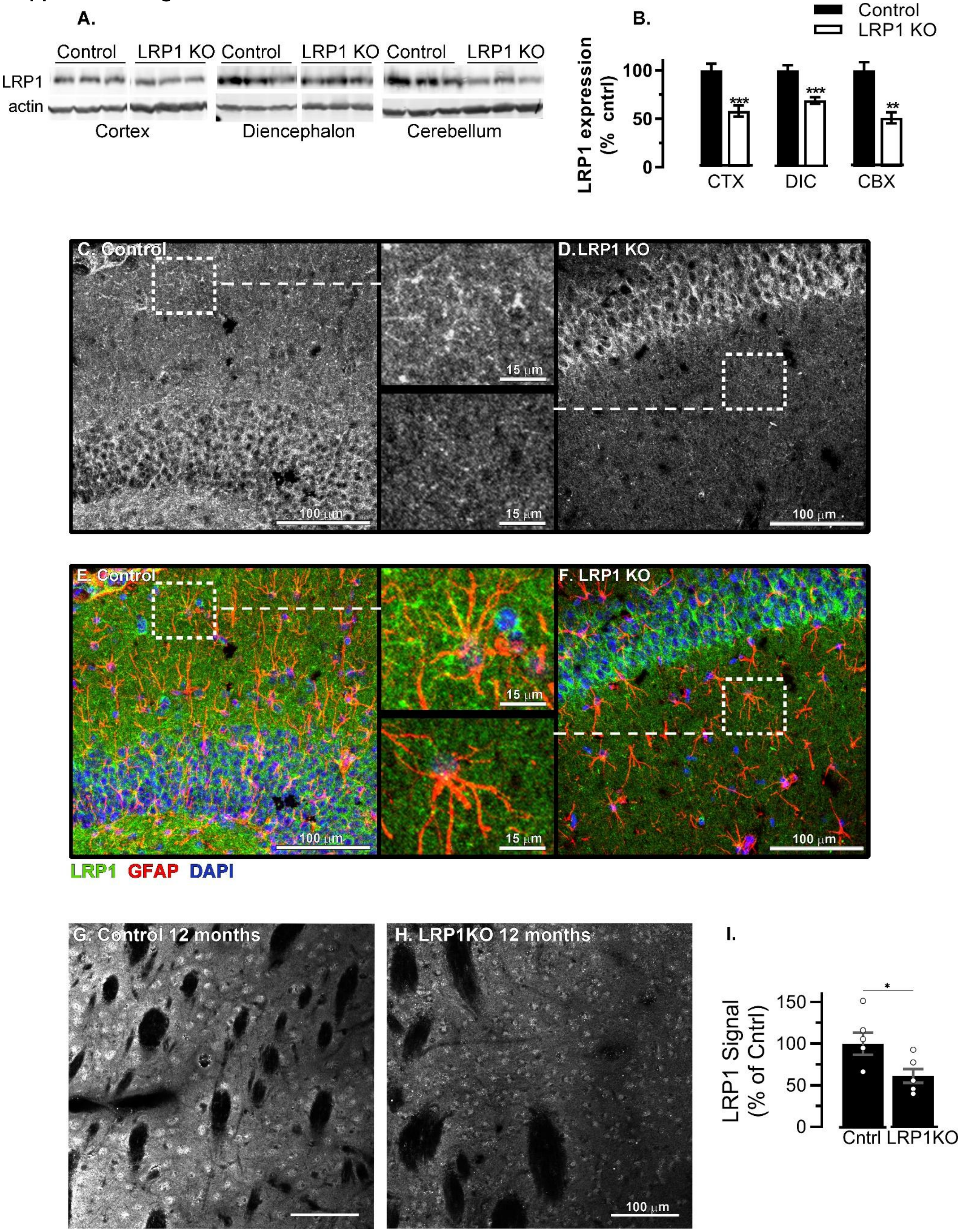

Figure S1: Total LRP1 expression by brain region in Control and LRP1KO adult mice. (A) Two-month-old mice were subjected to tamoxifen treatment, and LRP1 expression was assessed 2 weeks later in Control and LRP1KO mice. Representative western blot images of total LRP1 expression in brain tissue lysates harvested from the cortex, diencephalon, and cerebellum. **(B)** Averages±SEM of LRP1 expression from n=6 mice/group. LRP1 immunolabeling was performed in **(C)** Control and (**D)** LRP1KO mice. **(E,F)** Merged images showing labeling of LRP1 (green), GFAP (red) and nuclear DAPI (blue). **(C-F)** White squares outline regions of increased magnification inside the middle panels. Representative images are also shown of LRP1-immunolabelled striatum from 12-month old mice, 9 months after tamoxifen treatment in **(G)** Control and **(H)** LRP1KO mice. **(I)** Intensity of total LRP1 immunolabelling was measured in n=6 mice. **p<0.05, **p<0.01, ***p<0.001 compared to Control mice via Student's t test.*

Supplemental Figure 2:

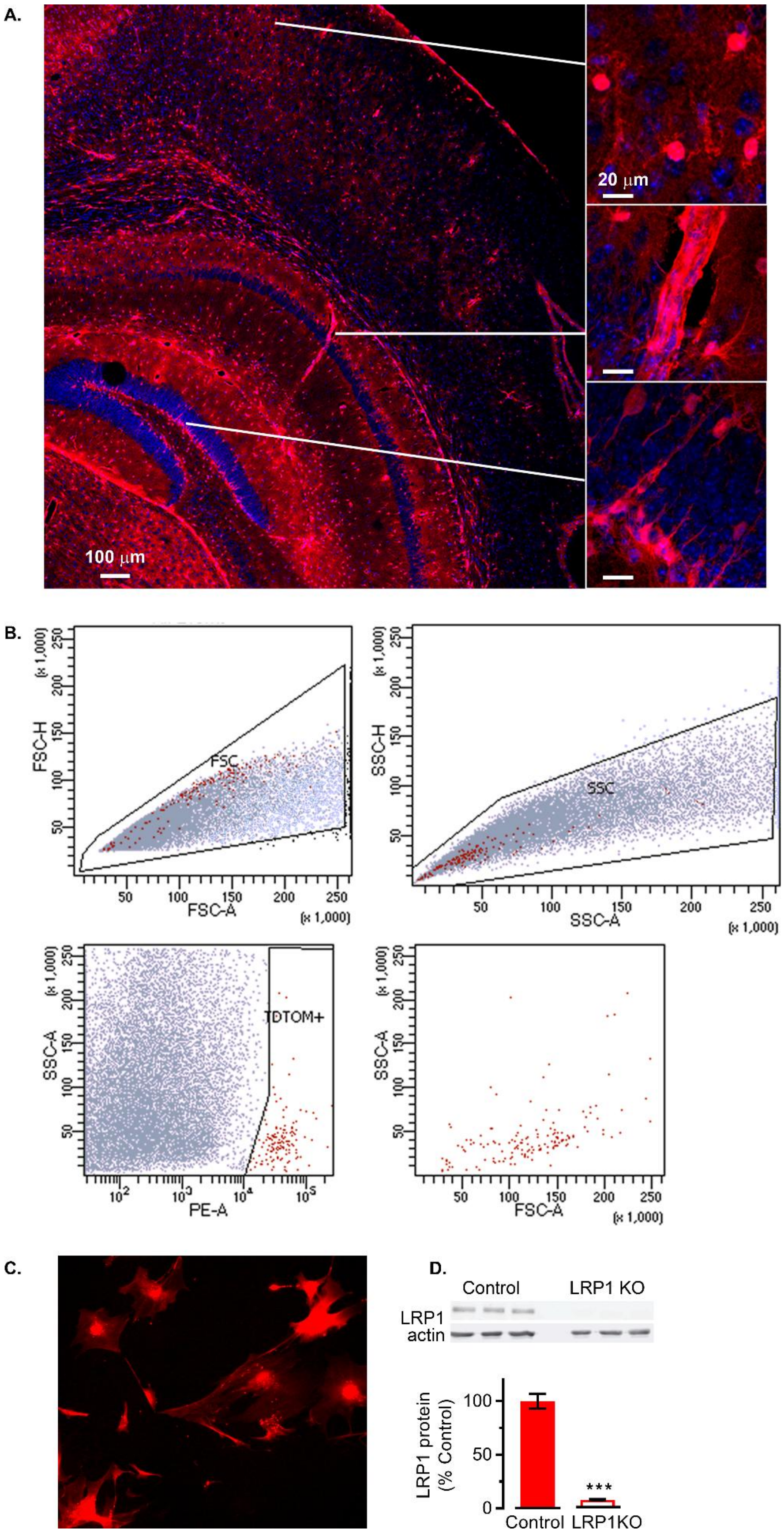

Figure S2: LRP1 expression in astrocytes of tomato-Control and tomato-LRP1KO mice. (A) In these mice, cells that express Cx30-Cre^{ERT2} are marked by the red fluorescent protein tdTomato, allowing visualization of astrocytes throughout the brain, including cortical astrocytes, astrocytes interacting with blood vessels, and in the hippocampus (inset). Cells harvested from the brains of 3-month-old mice (1 month post-tamoxifen) were sorted via FACS. **(B)** Scatter plots showing gates used to limit sorting to viable tdTomato positive cells, as shown in red. **(C)** Micrograph of viable tdTomato positive astrocytes sorted via FACS and grown in tissue culture. **(D)** Western blot analysis of LRP1 expression in primary astrocytes isolated from tomato-Control and tomato-LRP1KO mice. *Results are averages ±SEM of n=3 wells/genotype in 24-well plate from triplicate independent isolations. ***p<0.001 via Student's t test.*

Supplemental Figure 3:

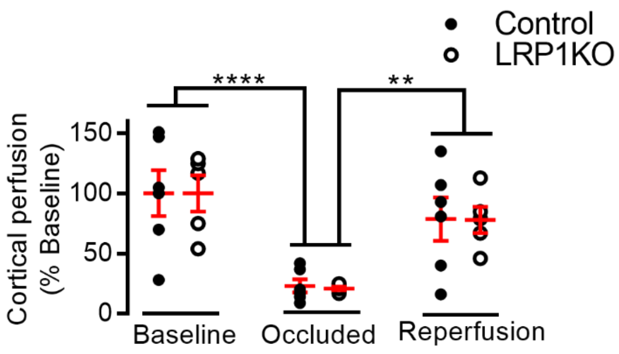

Figure S3: Laser Doppler readings of mice undergoing MCAO. Cortical blood flow was measured via laser Doppler probe on anesthetized mice prior to MCAO (baseline), during MCAO (occluded), and after removal of filament (reperfusion). Cortical blood flow is expressed as a percentage of baseline values. *Results are averages±SEM from n=5-6 mice/genotype. **p<0.01 and ****p<0.0001 via one-way ANOVA and paired Tukey's HSD*.

Supplemental Figure 4:

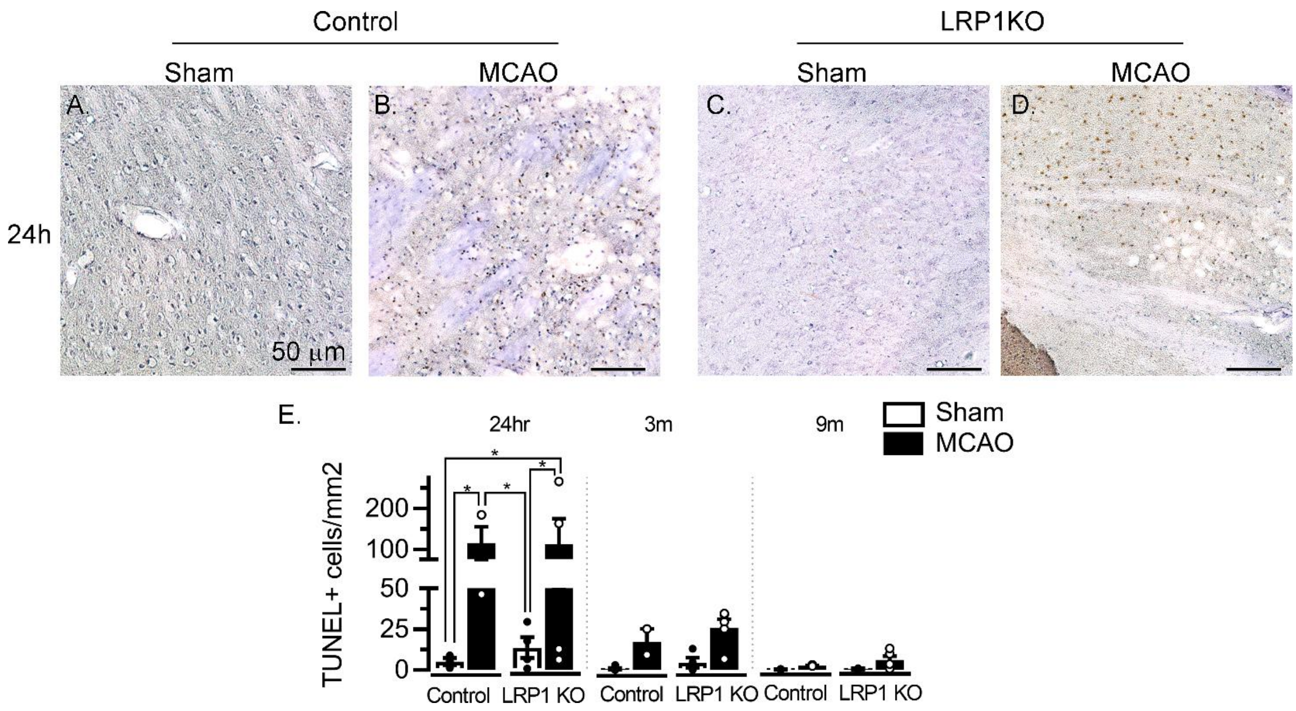

Figure S4: Apoptosis in striatum after MCAO. Apoptosis within the striatum was measured by staining apoptotic nuclei for TUNEL (brown nuclei). Representative images of striatal tissue 24 hours after MCAO counterstained with hematoxylin (blue) are shown for **(A)** Sham-treated Control, **(B)** MCAO-treated Control, **(C)** Sham-treated LRP1KO, and **(D)** MCAO-treated LRP1KO mice. **(E)** TUNEL+ cells within the striatal lesion were quantified as cells/mm^2 for 24 hours, 3 months, and 9 months post-surgery. *Results are pooled averages±SEM from 3-4 slices per mouse, with n=4-6 mice/group. *p<0.05 via two-way ANOVA followed by Tukey's HSD. Lack of asterisk indicates no significant differences.*

Supplemental Figure 5:

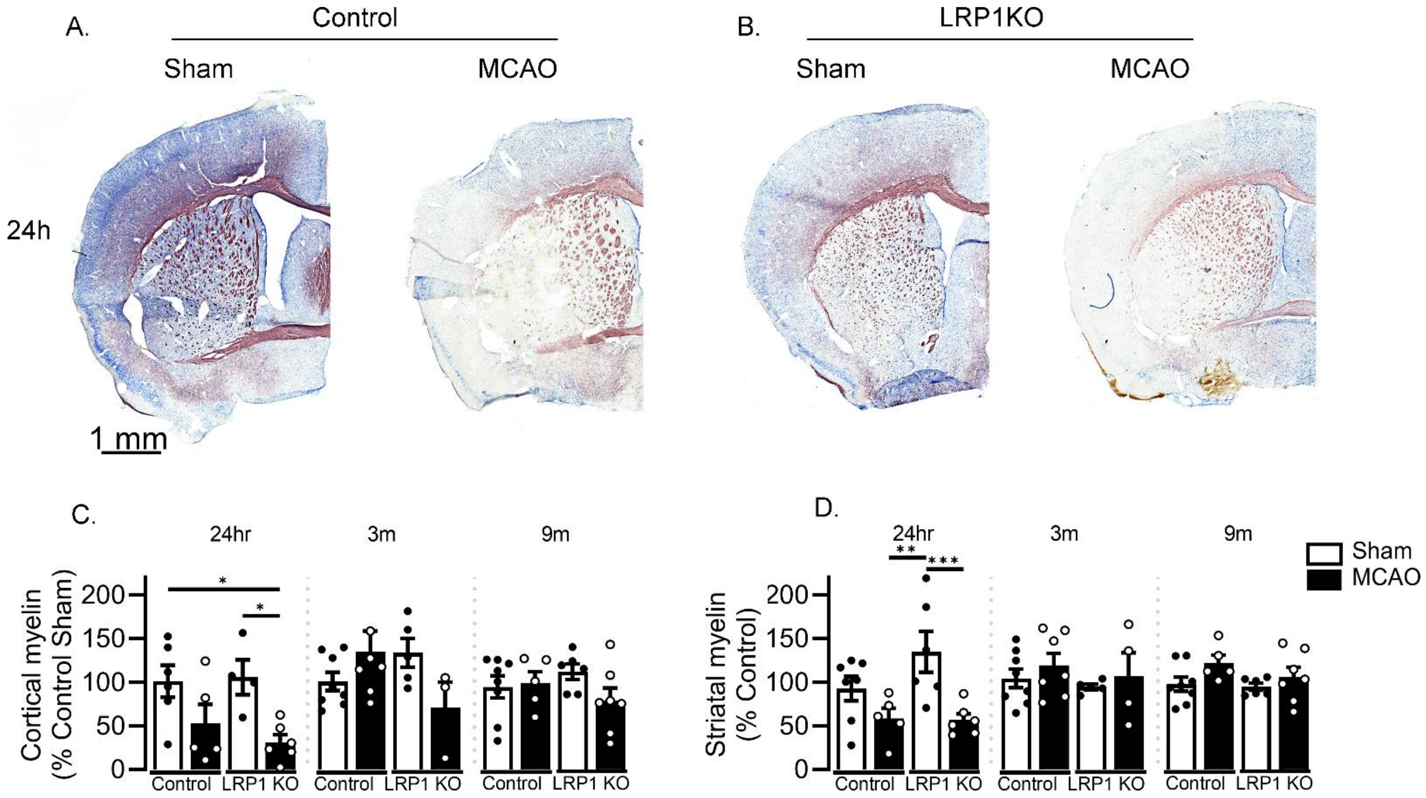

Figure S5: Myelination after MCAO. Myelin in harvested brain tissues from Control and LRP1KO mice was labelled using Black-Gold II staining at 24 hours, 3 months, and 9 months post-surgery. Representative images of mice 24 hours after surgery are shown for **(A)** Control and **(B)** LRP1KO mice. Myelination was quantified in **(C)** cortex and **(D)** striatum. *Results are pooled averages±SEM from 3-4 slices per mouse, with n=3-8 mice/group. *p<0.05, **p<0.01, ***p<0.001 via two-way ANOVA followed by Tukey's HSD.*

Supplemental Figure 6:

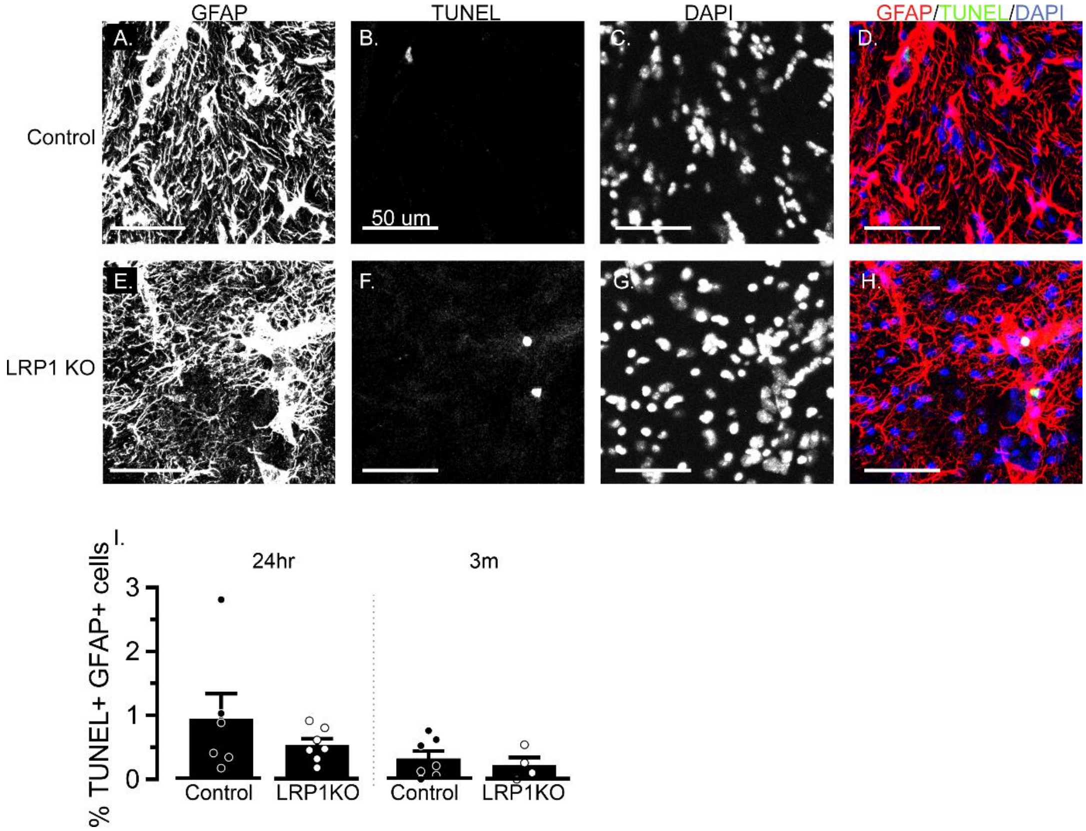

Figure S6: Astrocytic apoptosis in striatum after MCAO. Apoptosis of astrocytes in the striatum was measured by staining apoptotic nuclei for TUNEL and co-labeling with GFAP to visualize astrocytes. Representative images of striatal lesions at 3-months after MCAO are shown for **(A-D)** Control and **(E-H)** LRP1KO mice. **(I)** Apoptotic astrocytes are expressed as a percentage of total GFAP-positive cells for 24 hours, and 3 months post-surgery. *Results are pooled averages±SEM from 3-4 slices per mouse, with n=4-7 mice/group. Statistical significance via unpaired Student's t test.*

Supplemental Figure 7:

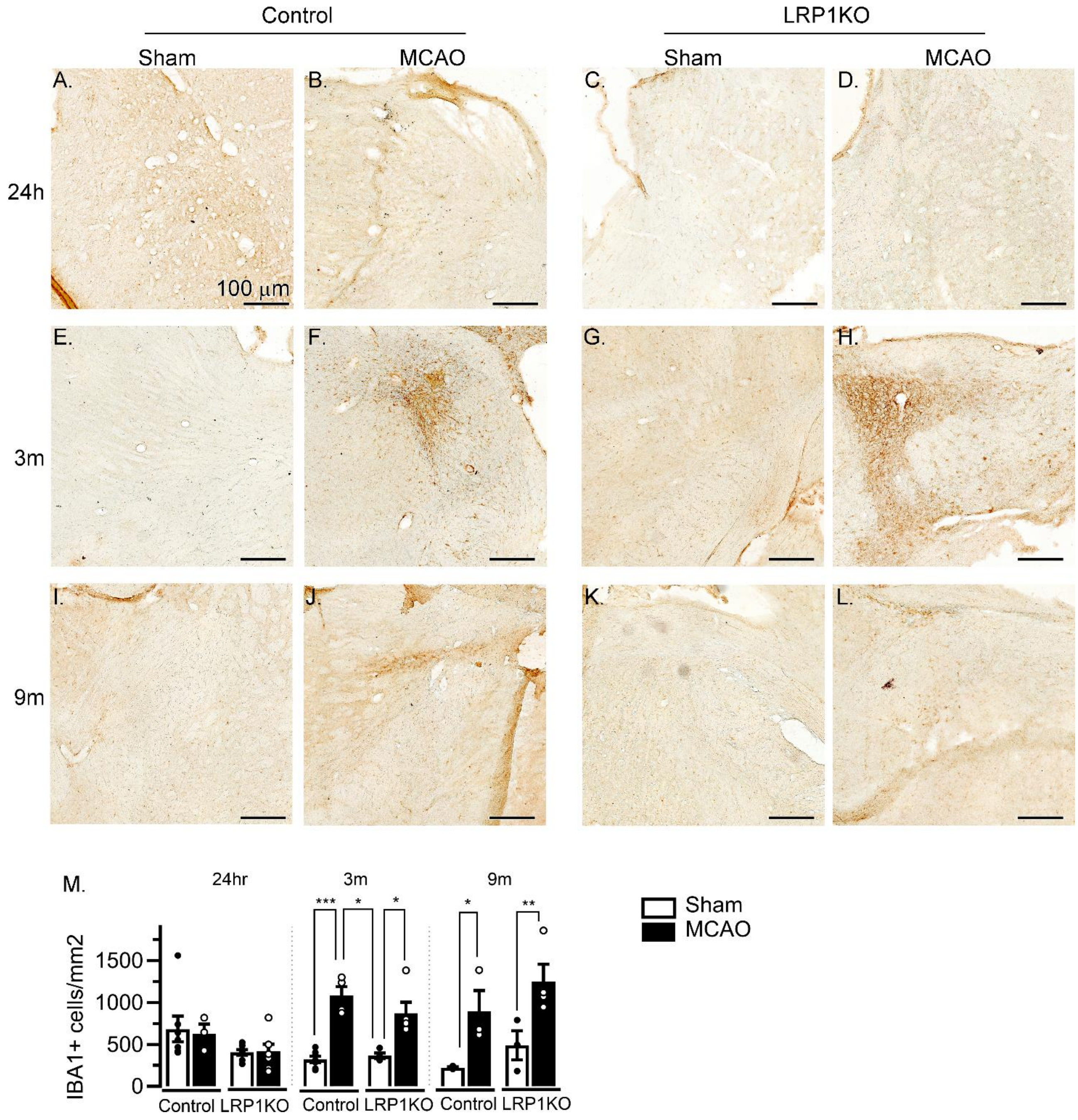

Figure S7: Microglial proliferation in the striatum after MCAO. Colorimetric immunolabeling of IBA1 was performed to measure microglia levels within the striatum of **(A, B, E, F, I, J)** Control or **(C, D, G, H, K, L)** LRP1KO mice at **(A-D)** 24-hours, **(E-H)** 3 months, or **(I-L)** 9 months after surgery. **(M)** Microglia were quantified as IBA1+ cells/mm^2 for 24 hr, 3 mon, and 9 mon post-surgery. *Results are expressed as averages±SEM pooled from 3-4 brain slices/mouse in n=6 mice/group. *p<0.05, **p<0.01, ***p<0.001 via two-way ANOVA followed by Tukey's HSD.*

Supplemental Figure 8:

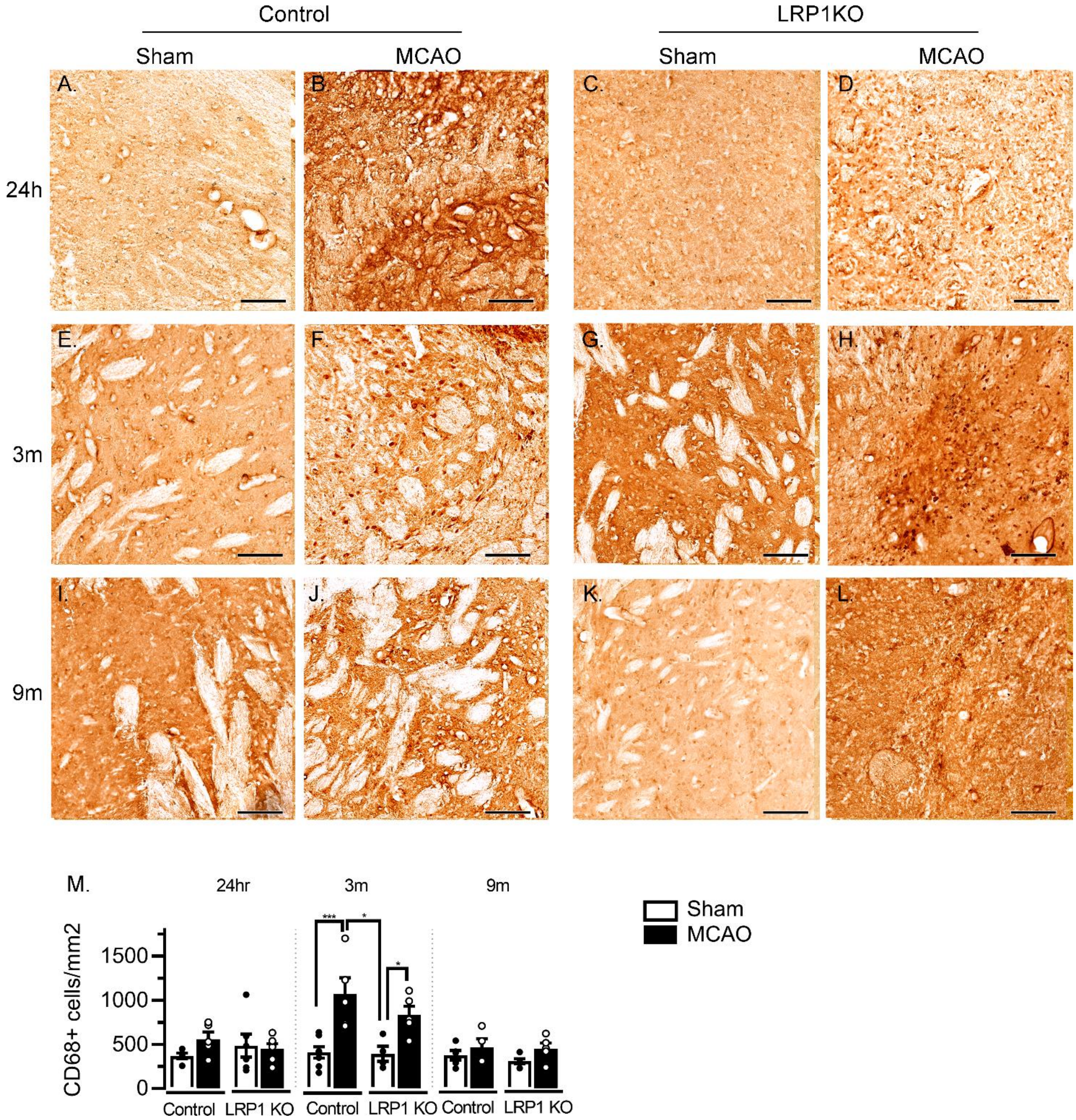

Figure S8: Microglial activation in the striatum after MCAO. Colorimetric immunolabeling of CD68 was performed to measure microglial activation within the striatum of **(A, D, E, F, I, J)** Control and **(C, D, G, H, K, L)** LRP1KO mice. Representative images are shown for **(A-D)** 24 hours, **(E-H)** 3 months, and **(I-L)** 9 months post-surgery (*scale bar=50 µm).* **(M)** Microglial activation was quantified as CD68+ cells/mm^2. *Results are expressed as averages±SEM pooled from 3-4 brain slices/mouse in n=6 mice/group. *p<0.05 and ***p<0.001 via two-way ANOVA followed by Tukey's HSD.*

Supplemental Figure 9:

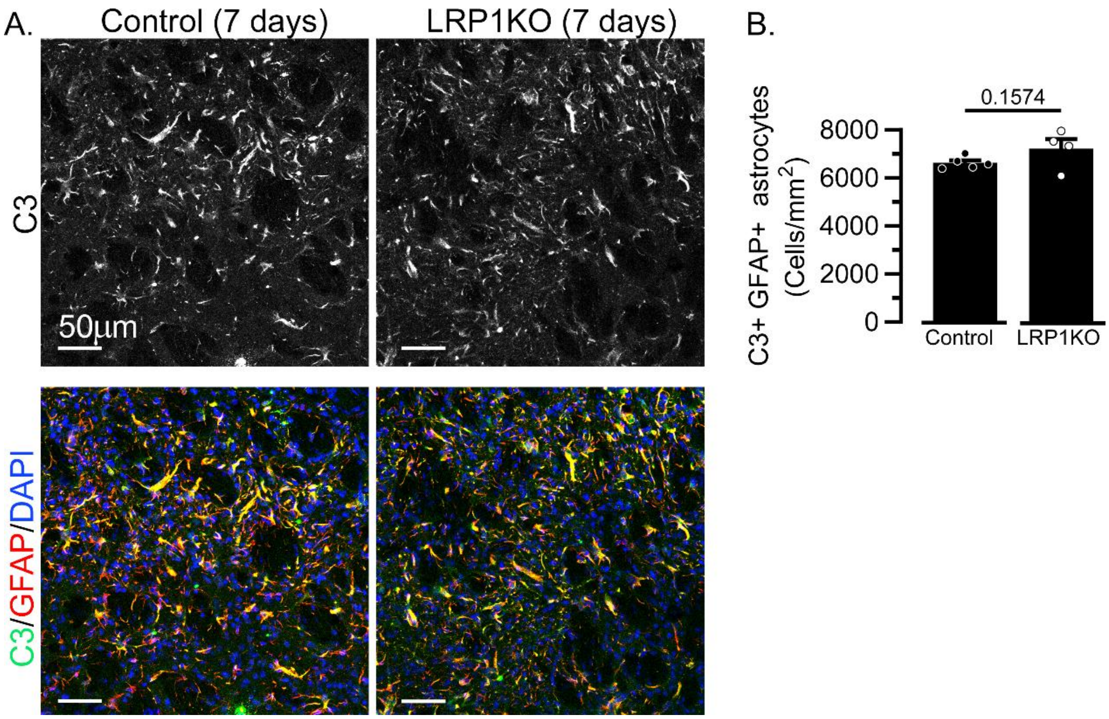

Figure S9: Full-length C3 expression in astrocytes 7 days after MCAO. Fluorescent immunolabeling for C3 and GFAP was performed in Control and LRP1KO mice. **(A)** Representative images showing C3 (top panels) and the merged image in the bottom panels. C3 is shown in green, GFAP is red, and DAPI-positive nuclei are blue. **(B)** The total density of C3+, GFAP+ astrocytes was determined. *Results are averages±SEM pooled from 3-6 images/mouse in n=4-6 mice/group*.
